# Vision-Assisted UAV Relay Triggering for Proactive Blockage Mitigation in Air–Ground Integrated mmWave V2X Networks

**DOI:** 10.3390/s26165180

**Published:** 2026-08-16

**Authors:** Yicheng Wang, Weiyan Chen, Luting Kong, Xiaoyang Wang, Weiwen Weng, Yang Liu, Yuehong Gao, Xin Zhang

**Affiliations:** 1 School of Information and Communication Engineering, Beijing University of Posts and Telecommunications, Beijing 100876, China; wangyicheng@bupt.edu.cn (Y.W.); yhgao@bupt.edu.cn (Y.G.); 2 China Mobile Research Institute, Beijing 100053, China; kongluting@chinamobile.com (L.K.); wangxiaoyangyjy@chinamobile.com (X.W.); wengweiwen@chinamobile.com (W.W.); liuyangaw@chinamobile.com (Y.L.)

**Keywords:** mmWave V2X communications, UAV relay triggering, blockage prediction, multi-camera perception, target association, template-guided image matching

## Abstract

Millimeter-wave (mmWave) vehicular-to-everything (V2X) links are highly vulnerable to sudden blockages in dense urban traffic. Since terrestrial roadside links can degrade rapidly, and alternative ground paths are often limited, maintaining reliable service with only ground networking resources remains challenging. To enhance link reliability by exploiting aerial relay resources in air–ground integrated networks, this paper proposes a vision-assisted unmanned aerial vehicle (UAV) relay triggering framework. The framework uses roadside multi-camera images to predict the future link state of a target vehicle and triggers a UAV decode-and-forward (DF) relay before the direct roadside-unit (RSU)–vehicle link becomes unreliable. To enable target-specific prediction, a template-guided image-matching module is developed to localize the target vehicle in multi-view images. The matched features are fused and temporally modeled to predict future LoS, NLoS, and Absent states, with the predicted NLoS probability further used to determine the UAV activation decision through a probability-based triggering policy. Simulation results on a 3D ray-tracing urban V2X dataset show that the proposed dual-view predictor achieves about 99% validation accuracy, compared with about 87% for the single-view baseline. The proposed relay triggering scheme reduces the outage probability from 15.08% for RSU-only transmission and 4.49% for reactive relaying to 0.76%, and improves the 5th-percentile rate from 11.72 Mbps to 22.49 Mbps over reactive relaying.

## 1. Introduction

Millimeter-wave (mmWave) communication is a key enabling technology for high-rate and low-latency vehicular-to-everything (V2X) services. By providing large available bandwidths, mmWave V2X can support data-intensive applications such as autonomous driving, cooperative perception, and intelligent transportation [1,2]. However, mmWave links are highly susceptible to beam misalignment and dynamic blockages caused by vehicles, roadside objects, and urban structures [3,4]. In dense traffic, sudden line-of-sight (LoS) interruptions can lead to severe received-power degradation, transmission delay, and service outage. Therefore, reliable mmWave V2X communication requires proactive blockage mitigation in addition to beam management.

Several methods have been studied to improve the robustness of mmWave vehicular communications, including multi-connectivity, resource allocation, relay selection, and beam management [5,6,7]. These methods enhance reliability by maintaining candidate links or switching to alternative transmission paths when the direct link becomes unreliable. However, redundant connectivity may introduce additional resource consumption, while reactive recovery may still suffer from response delay after link degradation.

Air–ground integrated V2X networks provide a promising paradigm for enhancing the reliability of blockage-prone vehicular communications. By jointly exploiting terrestrial RSUs and aerial UAV nodes, such networks can provide complementary communication resources beyond purely ground-based networking. In particular, UAVs introduce an additional aerial relay resource and can establish flexible air-to-ground paths when roadside links are blocked. Due to their three-dimensional mobility and deployment flexibility, UAVs have been widely studied as aerial relays for coverage extension, reliability enhancement, and Internet-of-Vehicles services [8,9,10]. However, continuously using the UAV relay introduces unnecessary relay transmissions and communication-resource occupation when the direct RSU–vehicle link is already reliable. Therefore, in air–ground integrated mmWave V2X networks, the UAV relay assistance should be triggered selectively and proactively according to the predicted reliability of the direct link.

Environmental sensing enables proactive relay triggering by predicting future link states before direct-link degradation occurs. LiDAR and point-cloud data have been used for blockage and link-quality prediction in high-frequency wireless systems [11,12]. Although such sensing modalities provide accurate geometric information, their deployment cost and sensing complexity may limit large-scale roadside deployment. In contrast, RGB cameras are low-cost, widely available, and easy to integrate with roadside infrastructure. Public vision-wireless and multi-modal sensing datasets, such as ViWi and DeepSense 6G, have further promoted learning-based beam prediction, blockage prediction, and sensing-assisted wireless communication research [13,14,15]. Visual information has been exploited for mmWave beam prediction, blockage prediction, and proactive handoff [16,17]. Recent studies have further applied computer vision to V2X beam alignment and LoS/blockage prediction [18,19,20]. However, most existing vision-aided methods focus on beam selection, link-state prediction, or handoff, while target-specific blockage prediction for UAV relay triggering remains less explored.

A key challenge in roadside vision-assisted V2X systems is target association. Roadside cameras usually observe multiple vehicles and blockers simultaneously, making it necessary to associate the target vehicle with its corresponding regions in multi-view images before blockage prediction. Existing methods often rely on external position information, beam-related cues, or detection results for target localization. However, GPS- or position-based localization may be inaccurate in GPS-denied or dense urban scenarios [21], and coordinate-to-image mapping errors may cause target mismatch in crowded traffic scenes. Template-guided matching and long-term tracking can locate a specified target in complex scenes [22,23,24]. This motivates image-matching-based target association, which identifies the target vehicle directly in the visual feature space.

Vision-aided UAV-assisted link selection or handover has been preliminarily studied in V2X systems [25]. However, the existing studies mainly focus on vehicle-side observations, link selection, or UAV trajectory control. Roadside target-aware multi-camera prediction for proactive UAV relay triggering remains insufficiently investigated. In particular, it is still challenging to associate the target vehicle across multi-view roadside images, predict its future link state, and trigger UAV decode-and-forward (DF) relay assistance before the direct RSU–vehicle link becomes unreliable.

To address these challenges, this paper proposes a target-aware multi-camera visual prediction and UAV relay triggering framework for air–ground integrated mmWave V2X networks. The framework exploits roadside visual perception to predict the future state of the target RSU–vehicle link and triggers UAV DF relaying before severe direct-link degradation occurs. A template-guided image-matching module is developed to localize the target vehicle in multi-view roadside images. The matched features are then fused across camera views and temporally modeled to predict future LoS, NLoS, and Absent states. The predicted NLoS probability is finally mapped to the UAV activation decision through a probability-based triggering policy. In this way, the framework forms a closed-loop perception–prediction–relay mechanism that connects terrestrial visual sensing with aerial relay assistance.

The main contributions of this paper are summarized as follows:

- We propose a vision-assisted UAV relay triggering framework for air–ground integrated mmWave V2X networks. The framework models the UAV as an aerial backup DF relay and uses *H*-slot-ahead visual prediction to determine relay activation. Communication-level metrics are introduced to characterize the tradeoff between outage reduction and UAV relay usage, thereby linking terrestrial visual perception with aerial relay-triggering performance.
- We develop a target-aware multi-camera visual prediction method for roadside V2X perception. A target vehicle template provides target-specific appearance information, and template-conditioned channel-wise correlation is performed between the projected template feature and each projected scene-view feature. The resulting target-aware features are fused across camera views and modeled by an LSTM module to predict future LoS, NLoS, and Absent states.
- We construct a 3D urban V2X simulation dataset with synchronized multi-view RGB observations and ray-tracing-based link-state labels. The trained predictor is compared with single-view, GPS/vision-based, and Transformer-based prediction baselines and is further integrated into the air–ground relay triggering process for comparison with RSU-only, reactive UAV relay, and oracle UAV relay schemes. The simulation results show that the proposed framework improves target-aware blockage prediction and substantially reduces outage probability under the considered simulation settings.

This perception–prediction–relay principle also suggests a potential extension to low-altitude urban air–ground networks. Beyond the considered RSU–vehicle link, a UAV with a reliable LoS link may serve as an aerial relay for another UAV whose communication link is blocked by buildings or other urban obstacles.

## 2. System Model

### 2.1. Air–Ground Integrated Vision-Assisted Scenario and Prediction Timeline

We consider an air-ground integrated mmWave V2X network in an urban road environment, where a terrestrial RSU serves a target vehicle, and a low-altitude UAV is available as an aerial backup relay. The terrestrial component includes the RSU–vehicle direct communication link and roadside sensing infrastructure, while the aerial component provides an additional UAV-assisted relay path when the terrestrial link becomes unreliable. Due to the high blockage sensitivity of mmWave propagation, the direct RSU–vehicle link may be obstructed by surrounding vehicles or roadside obstacles, resulting in severe service degradation. To mitigate such blockage-induced degradation, the UAV can be triggered as a backup decode-and-forward (DF) relay when the direct link is predicted to become unreliable.

**Figure 1:**
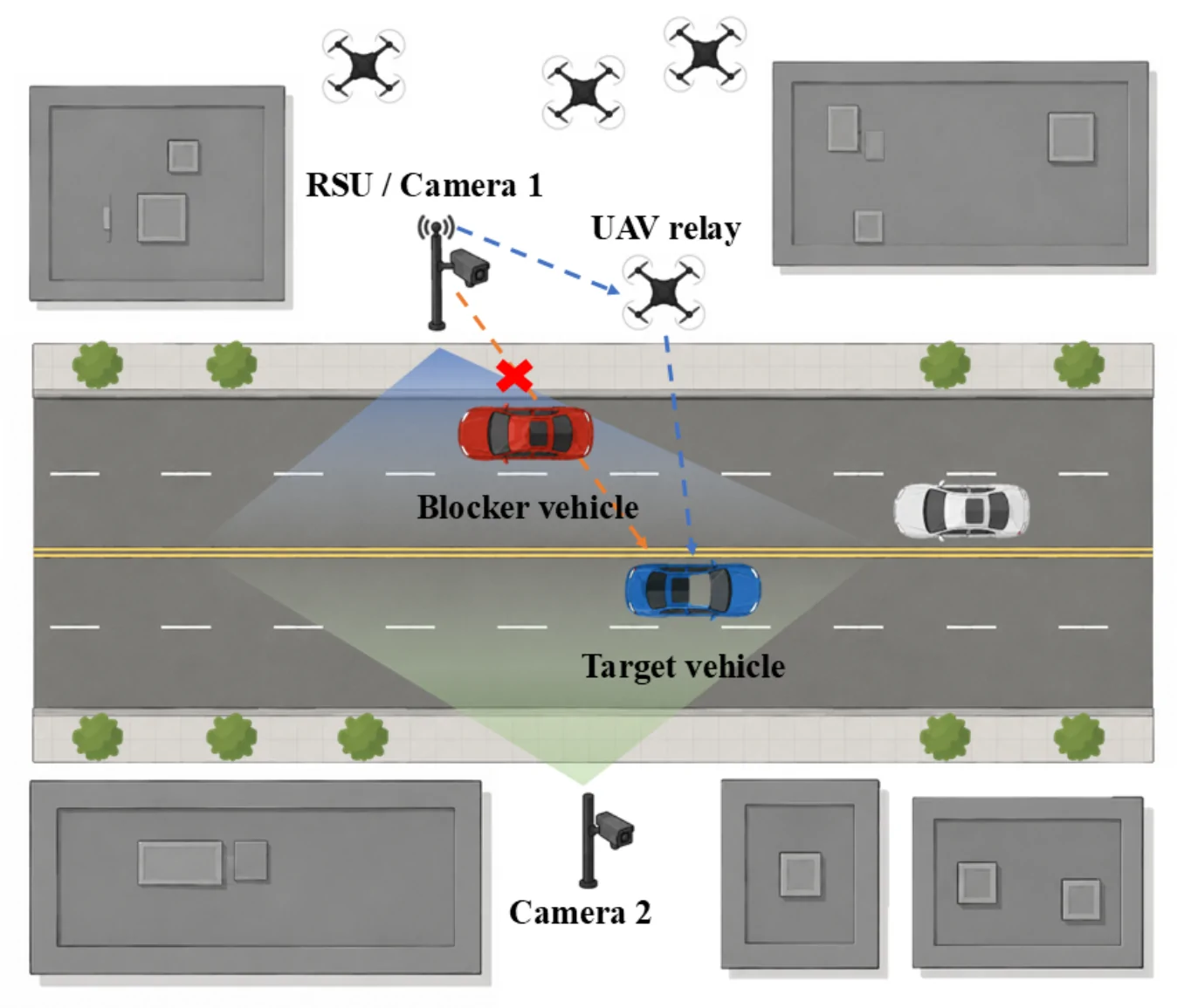
A top-down view of the simulated urban road environment. The orange dashed arrow denotes the blocked RSU–target-vehicle direct link, the red cross marks the blockage, the blue dashed arrows denote the UAV relay path, and the blue and green shaded regions indicate the fields of view of Camera 1 and Camera 2, respectively.

The roadside sensing infrastructure consists of two RGB cameras for environmental perception, one installed near the RSU and the other deployed on the opposite side of the road. The two cameras synchronously capture multi-view observations of the target vehicle, surrounding vehicles, and potential blockers. In addition, a pre-acquired template image U of the target vehicle is available to provide target-specific appearance information. In this way, terrestrial visual sensing is used to guide aerial relay activation, forming an air–ground integrated perception–prediction–relay scenario. The target-template-based visual association mechanism is detailed in Section 4. The considered air–ground integrated scenario and the corresponding communication and sensing links are illustrated in Figure 1.

The system operates in a time-slotted manner. Let t=1,2,…,T denote the slot index and Δt denote the slot duration. The visual module follows a sequence-to-one prediction structure. At each slot *t*, the most recent *r* consecutive multi-view image pairs are collected as

(1) $$X_{t}={\left\{\left(I_{\tau }^{\left(1\right)},I_{\tau }^{\left(2\right)}\right)\right\}}_{\tau =t-r+1}^{t},$$

where $I_{\tau }^{\left(m\right)}\in R^{H_{I}\times W_{I}\times 3}$ denotes the RGB image captured by camera m∈{1,2} at slot τ, and $H_{I}$ and $W_{I}$ are the image height and width, respectively. For compact notation, the target-specific visual input is denoted by

(2) $$Z_{t}=\left(X_{t},U\right),$$

where $X_{t}$ provides multi-view temporal scene observations, and $U\in R^{H_{U}\times W_{U}\times 3}$ denotes the target vehicle template with height $H_{U}$ and width $W_{U}$.

Let $s_{t}$ denote the direct RSU–vehicle link state at slot *t*, which is defined as

(3) $$s_{t}=\left\{\begin{array}{cc} 0, & \mathrm{LoS},\\ 1, & \mathrm{NLoS},\\ 2, & \mathrm{Absent}, \end{array}\right.$$

where LoS and NLoS indicate whether a line-of-sight propagation path exists between the RSU and the target vehicle, while Absent means that the target vehicle is outside the effective perception or service region. When $s_{t}=2$, the target vehicle is excluded from link-rate evaluation and relay triggering.

For the relay decision at service slot *t*, the visual module uses the information available *H* slots earlier to predict the link state at slot *t*:

(4) $$p_{t}^{\left(H\right)}=f_{\Theta }\left(Z_{t-H}\right),$$

where $p_{t}^{\left(H\right)}$ denotes the predicted probability vector of the link state at slot *t*. The corresponding hard prediction is obtained as ${\hat{s}}_{t}^{\left(H\right)}=\arg\;\max_{c}p_{t,c}^{\left(H\right)}$. The relay decision at slot *t* is made based on the predicted NLoS probability $p_{t,1}^{\left(H\right)}$ before the actual state $s_{t}$ is observed. The prediction horizon *H* represents the look-ahead interval of future link-state prediction and is distinct from the detection-to-activation response delay of the reactive scheme.

### 2.2. Communication and Relay Transmission Model

Let $q_{R}\in R^{3\times 1}$, $q_{V}\left(t\right)\in R^{3\times 1}$, and $q_{U}\in R^{3\times 1}$ denote the 3D positions of the terrestrial RSU, the target vehicle, and the aerial UAV relay, respectively. In the considered air–ground integrated network, the UAV is modeled as a fixed low-altitude backup relay hovering at a predefined road-corridor position. This setting isolates the relay-triggering problem from UAV trajectory control and allows the effect of visual blockage prediction on relay activation to be evaluated independently. When the target vehicle is within the service region, the terrestrial RSU–vehicle link is maintained as the direct transmission path, while UAV activation provides an additional aerial relay path.

The direct-link distance is given by

(5) $$d_{d}\left(t\right)=∥q_{R}-q_{V}\left(t\right)∥.$$

The large-scale path loss of the direct RSU–vehicle link is modeled according to the LoS/NLoS state as

(6) $$L_{d}\left(t\right)=L_{0}+10\alpha_{s_{t}}\log_{10}\left(\max\{d_{d}\left(t\right),1\}\right)+I\left\{s_{t}=1\right\}L_{N},$$

where $L_{0}=20\log_{10}\left(4\pi f_{c}/c\right)$ is the free-space path loss at the reference distance of 1 m, $f_{c}$ is the carrier frequency, *c* is the speed of light, $\alpha_{s_{t}}$ is the path-loss exponent determined by the link state, $L_{N}$ is the additional NLoS attenuation, and I{·} denotes the indicator function. Specifically, $\alpha_{s_{t}}=\alpha_{L}$ for the LoS state ($s_{t}=0$), and $\alpha_{s_{t}}=\alpha_{N}$ for the NLoS state ($s_{t}=1$).

The RSU employs a half-wavelength ULA and a DFT beam codebook. For receiver node x∈{V,U}, representing the target vehicle and the UAV, respectively, let $\Delta \phi_{x}\left(t\right)$ and $\Delta \theta_{x}\left(t\right)$ denote the relative azimuth and elevation offsets from the RSU panel boresight. The relative parabolic antenna element gain toward node *x* is modeled as

(7) $$G_{e,x}\left(t\right)=\max\left\{-12{\left(\frac{\Delta \phi_{x}\left(t\right)}{\phi_{3\mathrm{dB}}}\right)}^{2}-12{\left(\frac{\Delta \theta_{x}\left(t\right)}{\theta_{3\mathrm{dB}}}\right)}^{2},-A_{m}\right\},$$

where $\phi_{3\mathrm{dB}}$ and $\theta_{3\mathrm{dB}}$ are the horizontal and vertical half-power beamwidths, respectively, and $A_{m}$ is the maximum attenuation.

Let $a\left(u_{x}\left(t\right)\right)\in C^{N_{R}\times 1}$ denote the normalized ULA steering vector toward node *x*, where $u_{x}\left(t\right)$ denotes the normalized spatial frequency determined by the departure angle from the RSU to node *x*. Thus, $\alpha_{s_{t}}$ and $u_{x}\left(t\right)$ represent two distinct physical quantities, namely the path-loss exponent and the normalized spatial frequency, respectively. Specifically,

(8) $$a\left(u_{x}\left(t\right)\right)=\frac{1}{\sqrt{N_{R}}}{\left[1,e^{j\pi u_{x}\left(t\right)},\ldots ,e^{j\pi \left(N_{R}-1\right)u_{x}\left(t\right)}\right]}^{T}.$$

Let $f_{n}\in C^{N_{R}\times 1}$ denote the *n*-th unit-norm DFT beamforming vector. Under this normalization, ${\left|f_{n}^{H}a\left(u_{x}\left(t\right)\right)\right|}^{2}$ represents the normalized beam alignment gain, while the coherent array gain is accounted for by $N_{R}$. Therefore, the DFT beamforming gain toward node *x* is given by

(9) $$G_{b,x}\left(t\right)=10\log_{10}\left(N_{R}\max_{n}{\left|f_{n}^{H}a\left(u_{x}\left(t\right)\right)\right|}^{2}\right),$$

where $N_{R}$ is the number of RSU antenna elements.

The small-scale fading power gain is modeled as a unit-mean Nakagami-*m* random variable [26],

(10) g(t)∼Gamma(m,1/m),

where *m* is selected according to the link type. Accordingly, $g_{d}\left(t\right)$, $g_{\mathrm{RU}}\left(t\right)$, and $g_{\mathrm{UV}}\left(t\right)$ denote the small-scale fading power gains of the direct RSU–vehicle link, the RSU–UAV link, and the UAV–vehicle link, respectively. Considering the noise power, receiver noise figure, and implementation loss, the received SNR of the direct link is

(11) $$\gamma_{d}^{\mathrm{dB}}\left(t\right)=P_{R}+G_{\mathrm{RV}}\left(t\right)+10\log_{10}g_{d}\left(t\right)-L_{d}\left(t\right)-N_{\mathrm{th}}-F_{N}-L_{\mathrm{imp}},$$

where $P_{R}$ is the RSU transmit power, *B* is the system bandwidth, $N_{\mathrm{th}}=-174+10\log_{10}B$ is the thermal noise power in dBm, $F_{N}$ is the receiver noise figure, and $L_{\mathrm{imp}}$ denotes the implementation loss. With a practical spectral-efficiency cap, the achievable direct-link rate is

(12) $$R_{d}\left(t\right)=B\min\left\{\log_{2}\left(1+\gamma_{d}\left(t\right)\right),\eta_{\max}\right\},$$

where $\gamma_{d}\left(t\right)=10^{\gamma_{d}^{\mathrm{dB}}\left(t\right)/10}$, and $\eta_{\max}$ is the maximum spectral efficiency.

When the UAV relay is activated, an additional half-duplex DF relay path is established between the RSU and the target vehicle. The RSU–UAV and UAV–vehicle distances are

(13) $$d_{\mathrm{RU}}=∥q_{R}-q_{U}∥,$$

(14) $$d_{\mathrm{UV}}\left(t\right)=∥q_{U}-q_{V}\left(t\right)∥.$$

Since both the RSU and the UAV relay are fixed in the considered setup, $d_{\mathrm{RU}}$ is time-invariant, whereas $d_{\mathrm{UV}}\left(t\right)$ varies with the vehicle position. For the nominal communication evaluation, the UAV relay links are modeled as LoS-dominant air-to-ground channels, and their path losses are given by

(15) $$L_{\mathrm{RU}}=L_{0}+10\alpha_{L}\log_{10}\left(\max\{d_{\mathrm{RU}},1\}\right),$$

(16) $$L_{\mathrm{UV}}\left(t\right)=L_{0}+10\alpha_{L}\log_{10}\left(\max\{d_{\mathrm{UV}}\left(t\right),1\}\right).$$

The RSU–UAV hop uses the RSU DFT beamforming gain toward the UAV, whereas the UAV–vehicle hop uses the UAV transmit antenna gain. The received SNRs of the two relay hops are given by

(17) $$\gamma_{\mathrm{RU}}^{\mathrm{dB}}\left(t\right)=P_{R}+G_{\mathrm{RU}}\left(t\right)+10\log_{10}g_{\mathrm{RU}}\left(t\right)-L_{\mathrm{RU}}-N_{\mathrm{th}}-F_{N}-L_{\mathrm{imp}},$$

(18) $$\gamma_{\mathrm{UV}}^{\mathrm{dB}}\left(t\right)=P_{U}+G_{U}+10\log_{10}g_{\mathrm{UV}}\left(t\right)-L_{\mathrm{UV}}\left(t\right)-N_{\mathrm{th}}-F_{N}-L_{\mathrm{imp}},$$

where $P_{U}$ is the UAV transmit power, $G_{U}$ is the UAV transmit antenna gain, and $\gamma_{i}\left(t\right)=10^{\gamma_{i}^{\mathrm{dB}}\left(t\right)/10}$ for i∈{RU,UV}. To assess the sensitivity of the conclusions to this LoS-dominant assumption, a probabilistic LoS/NLoS extension of the two A2G hops is further considered in Section 6.2.

The achievable rates of the RSU–UAV and UAV–vehicle hops are

(19) $$R_{\mathrm{RU}}\left(t\right)=B\min\left\{\log_{2}\left(1+\gamma_{\mathrm{RU}}\left(t\right)\right),\eta_{\max}\right\},$$

(20) $$R_{\mathrm{UV}}\left(t\right)=B\min\left\{\log_{2}\left(1+\gamma_{\mathrm{UV}}\left(t\right)\right),\eta_{\max}\right\}.$$

For half-duplex DF relaying, the end-to-end relay rate is limited by the weaker hop and is given by

(21) $$R_{\mathrm{rel}}\left(t\right)=\frac{1}{2}\min\left\{R_{\mathrm{RU}}\left(t\right),R_{\mathrm{UV}}\left(t\right)\right\}.$$

The factor 1/2 explicitly accounts for the two orthogonal transmission phases required by half-duplex DF relaying. In contrast, the direct RSU–vehicle transmission does not require a second relay phase and therefore does not incur this half-duplex penalty. Thus, the relay rate already includes its spectral-efficiency loss relative to direct transmission.

Let $a_{t}\in \left\{0,1\right\}$ denote the UAV activation variable, where $a_{t}=1$ indicates that UAV relay assistance is triggered. Since relay activation provides an auxiliary path without disabling direct transmission, the effective service rate is modeled as

(22) $$R\left(t\right)=\left\{\begin{array}{cc} R_{d}\left(t\right), & a_{t}=0,\\ \max\left\{R_{d}\left(t\right),R_{\mathrm{rel}}\left(t\right)\right\}, & a_{t}=1. \end{array}\right.$$

Thus, the activated UAV relay offers an additional transmission option while preserving the direct link when it provides a higher rate.

## 3. Problem Formulation

Based on the system model above, this work aims to develop a visual prediction framework for proactive UAV relay triggering in air–ground integrated mmWave V2X networks. The predictor estimates the future blockage state of the target RSU–vehicle link, and the resulting prediction probability is used to trigger UAV relay assistance before severe direct-link degradation occurs.

For a visual prediction model parameterized by Θ, the predicted probability vector for service slot *t* is denoted by

(23) $$p_{t}^{\left(H\right)}=f_{\Theta }\left(Z_{t-H}\right),$$

where $p_{t}^{\left(H\right)}=\left[p_{t,0}^{\left(H\right)},p_{t,1}^{\left(H\right)},p_{t,2}^{\left(H\right)}\right]$ contains the predicted probabilities of the future LoS, NLoS, and Absent states, respectively.

The UAV relay activation decision is determined according to the predicted NLoS probability as

(24) $$a_{t}\left(\Theta ,\tau \right)=I\left\{p_{t,1}^{\left(H\right)}\geq \tau \right\},$$

where τ∈[0,1] denotes the NLoS-probability threshold.

Given the resulting relay decision, the service rate is

(25) $$R_{\Theta ,\tau }\left(t\right)=R\left(t\right){|}_{a_{t}=a_{t}\left(\Theta ,\tau \right)},$$

where R(t) follows the direct/relay transmission model defined in Section 2.

Let $R_{\mathrm{th}}$ denote the minimum required service rate of the target vehicle. Since relay triggering and rate evaluation are meaningful only when the target vehicle is within the effective service region and sufficient historical visual observations are available, the effective evaluation slot set is defined as

(26) $$T_{\mathrm{eff}}=\left\{t|s_{t}\in \left\{0,1\right\},\;t\geq H+r\right\}.$$

The average outage probability over $T_{\mathrm{eff}}$ is defined as

(27) $$P_{\mathrm{out}}\left(\Theta ,\tau \right)=\frac{1}{|T_{\mathrm{eff}}|}\sum_{t\in T_{\mathrm{eff}}}I\left\{R_{\Theta ,\tau }\left(t\right)<R_{\mathrm{th}}\right\},$$

where $|T_{\mathrm{eff}}|$ denotes the number of effective evaluation slots.

The average UAV activation ratio is defined as

(28) $$\eta_{\mathrm{UAV}}\left(\Theta ,\tau \right)=\frac{1}{|T_{\mathrm{eff}}|}\sum_{t\in T_{\mathrm{eff}}}a_{t}\left(\Theta ,\tau \right).$$

This metric represents the fraction of service slots in which UAV relay resources are occupied and is used to characterize the relay-usage overhead of the triggering policy.

The outage probability and UAV activation ratio are treated as communication-level evaluation metrics rather than as direct training objectives of the visual predictor. The prediction network is trained using the supervised cross-entropy loss described in Section 4. After training, the predicted NLoS probability is converted into the relay decision through the threshold τ, and the resulting outage probability and UAV activation ratio are used to evaluate the reliability–relay-usage tradeoff.

The relay decision is target-specific, whereas practical visual scenes may contain multiple vehicles and dynamic blockers. If the target vehicle is not accurately associated with the visual observations, the predictor may focus on an incorrect vehicle or lose target-related blockage information. This issue can be further aggravated when external localization cues, such as GPS positions or beamforming results, are inaccurate in urban road environments. Therefore, the following section develops a target-aware multi-camera prediction framework for robust future blockage prediction and proactive UAV relay triggering.

## 4. Proposed Solution

### 4.1. Framework Overview

**Figure 2:**
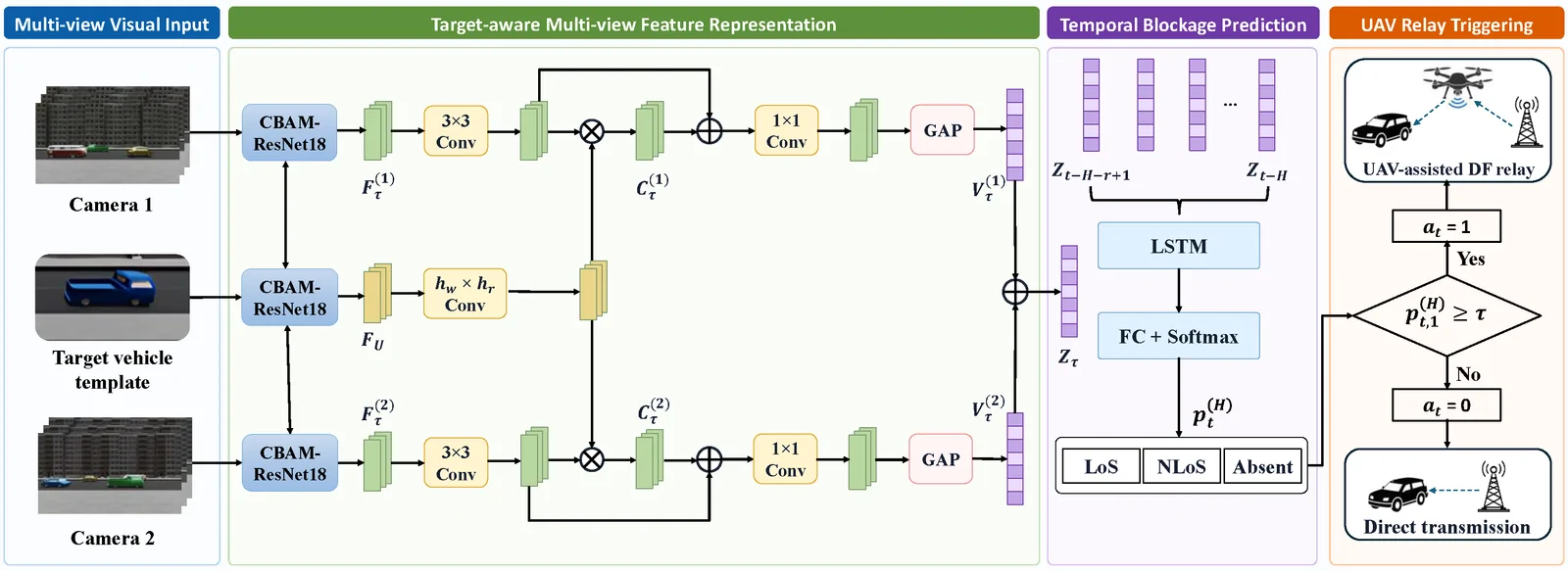
Overall framework of the proposed target-aware visual prediction and UAV relay triggering method.

Figure 2 illustrates the proposed target-aware visual prediction and UAV relay triggering framework. The framework contains three main stages: target-aware multi-view feature representation, multi-camera temporal blockage prediction, and prediction-assisted UAV relay triggering. In the first stage, target-related visual features are extracted from the multi-view image sequence and the target vehicle template. In the second stage, their temporal evolution is modeled to predict the future RSU–vehicle link state. In the third stage, the predicted NLoS probability is mapped to the UAV relay activation decision through a probability-based triggering policy.

For the relay decision at service slot *t*, the visual input is $Z_{t-H}=\left(X_{t-H},U\right)$, where $X_{t-H}$ denotes the multi-view image sequence observed *H* slots before slot *t*, and U is the target vehicle template. The prediction network produces

(29) $$p_{t}^{\left(H\right)}=f_{\Theta }\left(Z_{t-H}\right),$$

where $p_{t}^{\left(H\right)}$ contains the predicted probabilities of the future LoS, NLoS, and Absent states. The predicted NLoS probability is then used by the relay triggering policy to determine the UAV activation decision according to the selected operating threshold.

### 4.2. Target-Aware Multi-View Feature Representation

For each slot τ, the two RGB images $I_{\tau }^{\left(1\right)}$ and $I_{\tau }^{\left(2\right)}$ provide multi-view scene observations, while the template U provides target-specific appearance information. This module enhances target-related visual cues in each camera view and fuses them into a compact multi-view representation for temporal prediction.

A CBAM-enhanced ResNet18 is adopted as the shared visual backbone [27,28]. The fully connected layer is removed to preserve spatial feature maps for target-template correlation, while the CBAM blocks enhance informative channel and spatial responses in the scene. For camera view m∈{1,2}, the scene feature map is extracted as

(30) $$F_{\tau }^{\left(m\right)}=\phi_{b}\left(I_{\tau }^{\left(m\right)}\right),$$

where $\phi_{b}\left(\cdot \right)$ denotes the shared backbone. The target template feature is extracted by the same backbone:

(31) $$F_{U}=\phi_{b}\left(U\right).$$

This weight-sharing design maps the scene images and the target template into a common feature space while reducing the number of model parameters.

To obtain target-aware features in each view, the scene and template features are first transformed by learnable convolutional projection layers, after which channel-wise correlation is performed between the projected features:

(32) $$C_{\tau }^{\left(m\right)}=\psi_{s}\left(F_{\tau }^{\left(m\right)}\right)⊙\psi_{u}\left(F_{U}\right),\;m\in \left\{1,2\right\},$$

where $\psi_{s}\left(\cdot \right)$ and $\psi_{u}\left(\cdot \right)$ are learnable convolutional projection layers. The template projection produces a target-specific channel descriptor, which is spatially broadcast over the projected scene feature. The operator ⊙ denotes the resulting channel-wise multiplication. Specifically, the correlation feature is computed as

(33) $${\left[C_{\tau }^{\left(m\right)}\right]}_{c,i,j}={\left[\psi_{s}\left(F_{\tau }^{\left(m\right)}\right)\right]}_{c,i,j}{\left[\psi_{u}\left(F_{U}\right)\right]}_{c}.$$

This operation performs template-conditioned feature correlation independently along each channel through element-wise multiplication. The correlation operation itself introduces no additional trainable parameters, whereas the preceding convolutional projection layers are learnable. The resulting correlation feature emphasizes scene representations associated with the appearance characteristics of the target vehicle. Unlike position-guided association, this visual correlation is computed directly in the image feature space, reducing the dependence on external localization cues.

Since blockage prediction also depends on surrounding vehicles and potential blockers, the target-correlation feature is fused with the projected scene feature:

(34) $${\tilde{F}}_{\tau }^{\left(m\right)}=\rho_{m}\left(\psi_{s}\left(F_{\tau }^{\left(m\right)}\right)⊕C_{\tau }^{\left(m\right)}\right),\;m\in \left\{1,2\right\},$$

where ⊕ denotes channel-wise concatenation, and $\rho_{m}\left(\cdot \right)$ denotes a learnable 1×1 convolutional fusion layer. This fusion preserves both target-specific association cues and blockage-related scene context.

Global average pooling is then applied to obtain a compact feature vector for each camera view:

(35) $$v_{\tau }^{\left(m\right)}=\mathrm{GAP}\left({\tilde{F}}_{\tau }^{\left(m\right)}\right),\;m\in \left\{1,2\right\}.$$

The two view-specific features are concatenated to form the multi-view target-aware representation at slot τ:

(36) $$z_{\tau }=v_{\tau }^{\left(1\right)}⊕v_{\tau }^{\left(2\right)}.$$

The feature vector $z_{\tau }$ captures target appearance, multi-view scene observations, and blockage-related context and is used for subsequent temporal blockage prediction.

### 4.3. Multi-Camera Temporal Blockage Prediction

For the relay decision at service slot *t*, the visual input $Z_{t-H}$ is first converted into a sequence of target-aware feature vectors. Since $X_{t-H}$ contains the most recent *r* multi-view image pairs, the temporal feature sequence is written as

(37) $$H_{t}^{\left(H\right)}={\left\{z_{\tau }\right\}}_{\tau =t-H-r+1}^{t-H},$$

where $z_{\tau }$ is the multi-view target-aware feature vector extracted at slot τ. The prediction horizon *H* provides a time margin for proactive relay triggering before the actual link degradation occurs.

To model the temporal evolution of the target vehicle and potential blockers, the feature sequence $H_{t}^{\left(H\right)}$ is fed into an LSTM network:

(38) $$h_{t}^{\left(H\right)}=\mathrm{LSTM}\left(H_{t}^{\left(H\right)}\right),$$

where $h_{t}^{\left(H\right)}$ denotes the temporal hidden representation used to predict the link state at service slot *t*.

The hidden representation is then mapped to a state probability vector:

(39) $$p_{t}^{\left(H\right)}=\mathrm{softmax}\left(W_{o}h_{t}^{\left(H\right)}+b_{o}\right),$$

where $p_{t}^{\left(H\right)}=\left[p_{t,0}^{\left(H\right)},p_{t,1}^{\left(H\right)},p_{t,2}^{\left(H\right)}\right]$ contains the probabilities of LoS, NLoS, and Absent states, respectively, and $W_{o}$ and $b_{o}$ are trainable parameters. The predicted future link state is obtained as

(40) $${\hat{s}}_{t}^{\left(H\right)}=\arg\max_{c\in \{0,1,2\}}p_{t,c}^{\left(H\right)}.$$

For communication-oriented relay triggering, we further use $p_{t,1}^{\left(H\right)}$, i.e., the predicted probability of the future NLoS state, as a continuous blockage-confidence score. This allows the relay operating point to be adjusted through a probability threshold rather than relying only on the hard three-class prediction.

During training, the prediction network is optimized using ground-truth future link-state labels. For a training set D, the cross-entropy loss is defined as

(41) $$L_{\mathrm{CE}}=-\frac{1}{\left|D\right|}\sum_{(t,s_{t})\in D}\sum_{c=0}^{2}I\left\{s_{t}=c\right\}\log p_{t,c}^{\left(H\right)}.$$

This loss trains the predictor to map target-aware temporal observations to future link states. During inference, the trained predictor provides the future link-state probabilities, among which the predicted NLoS probability is used for UAV relay triggering.

### 4.4. Prediction-Assisted UAV Relay Triggering

The predicted future NLoS probability is converted into the UAV activation decision through a probability-threshold triggering policy:

(42) $$a_{t}\left(\Theta ,\tau \right)=I\left\{p_{t,1}^{\left(H\right)}\geq \tau \right\},$$

where τ∈[0,1] denotes the NLoS-probability threshold. A lower threshold increases the sensitivity to potential blockage and therefore tends to improve NLoS recall, but it may also introduce additional false-positive relay activations. Conversely, a higher threshold reduces unnecessary relay usage at the risk of missing future blockage events. Therefore, τ determines the operating tradeoff between blockage detection and relay usage. The operating threshold is selected on the validation set by maximizing the NLoS F1 score and is subsequently fixed for the communication-level evaluation.

Given $a_{t}\left(\Theta ,\tau \right)$, the resulting service rate is

(43) $$R_{\Theta ,\tau }\left(t\right)=R\left(t\right){|}_{a_{t}=a_{t}\left(\Theta ,\tau \right)}.$$

When relay assistance is triggered, the UAV provides an auxiliary path, and the effective rate follows the direct/relay selection model defined in the system model. Therefore, the proposed policy enhances blockage robustness while limiting unnecessary UAV activation when the predicted NLoS probability is low.

## 5. Experimental Setup

### 5.1. Dataset and Simulation Settings

**Figure 3:**
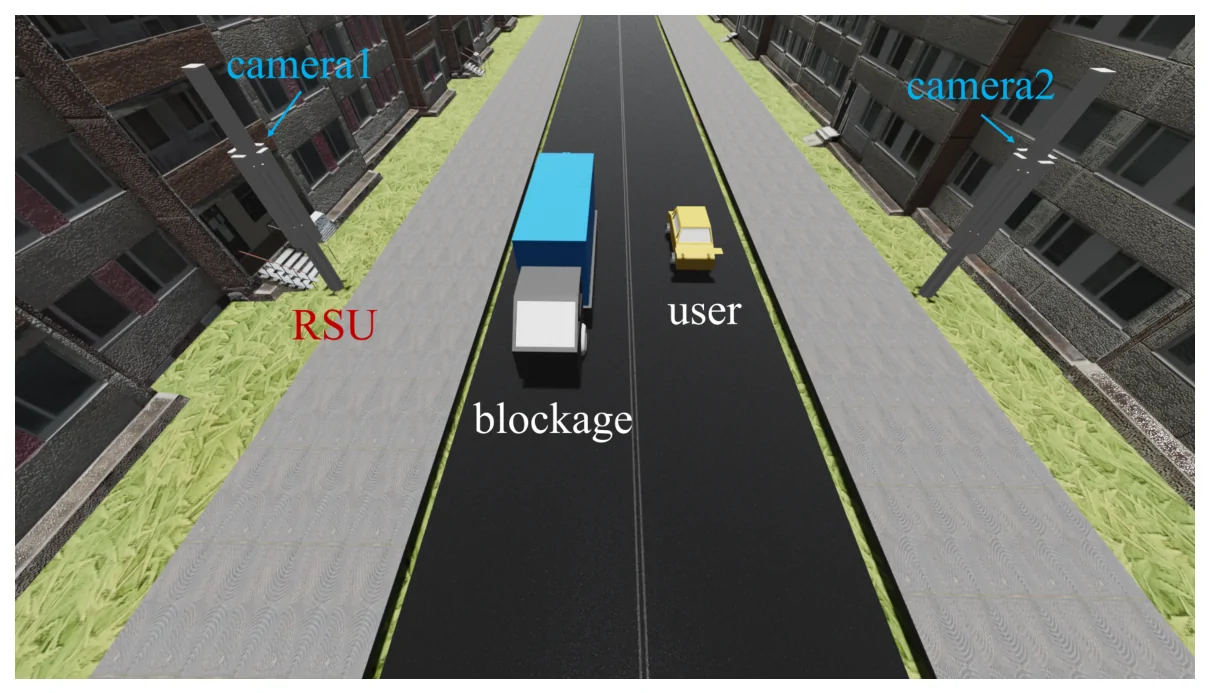
Simulated urban road scenario with two RGB cameras for vision-assisted blockage prediction.

**Figure 4:**
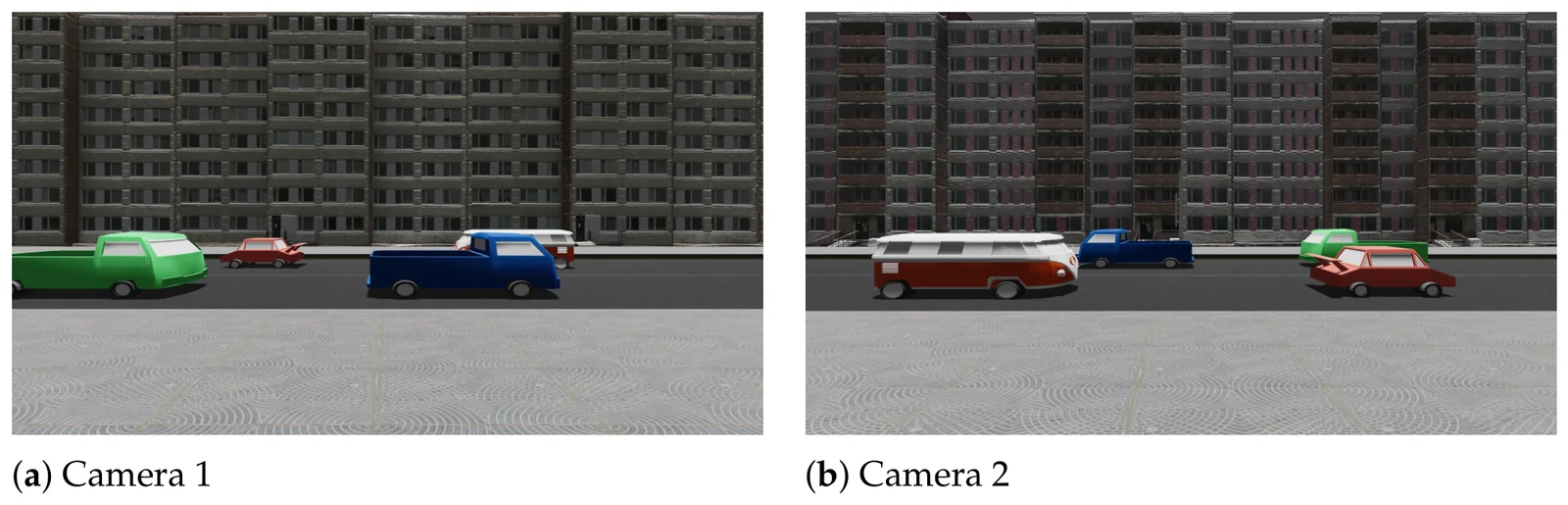
Representative RGB images captured by the two cameras.

To evaluate the proposed vision-assisted UAV relay triggering framework, we construct a 3D urban road scenario. The scenario is used to generate multi-view visual observations and wireless link-state labels. As illustrated in Figure 3, an RSU is deployed along the road to serve vehicular users. Two RGB cameras are used for environmental perception. One camera is installed near the RSU, while the other is deployed on the opposite side of the road to extend the visual coverage and provide a complementary viewpoint. Representative RGB images captured by the two cameras are shown in Figure 4.

The two RGB cameras synchronously capture the target vehicle, surrounding vehicles, and potential blockers. Meanwhile, ray-tracing-based channel simulation is performed to determine the direct RSU–vehicle link state. Each target vehicle is labeled as LoS, NLoS, or Absent according to its propagation condition and whether it is within the effective perception and service region. These labels are used as the ground truth for future blockage prediction.

The dataset contains 5000 time-sequenced scene samples, each including synchronized multi-view RGB image sequences and link-state labels for vehicles in the scene. The simulated traffic contains 10 vehicle instances covering four vehicle types, with variations in traffic configuration, vehicle motion, speed, relative position, and blockage relationships across different scene sequences. Each scene may contain multiple vehicles, whereas each prediction task focuses on one specific target vehicle. Therefore, target-specific instances are generated by selecting different vehicles from the same scene sequence. For each instance, a pre-acquired target vehicle template is paired with the corresponding multi-view scene sequence to support target-aware visual association. This process yields more than 15,000 valid target-specific prediction instances.

The training and validation sets are split at the scene-sequence level using an 80–20% ratio, resulting in approximately 12,000 training instances and 3000 validation instances. All target-specific instances derived from the same scene sequence are assigned to the same subset, thereby avoiding direct sequence-level leakage between training and validation data. The current evaluation uses the same road geometry and camera deployment for both subsets.

For communication-level evaluation, the trained visual predictor is integrated into the relay triggering process to assess its impact on link reliability. All schemes are evaluated under the same simulation settings for fair comparison.

### 5.2. Communication and Training Parameters

The main communication simulation parameters are summarized in Table 1. Unless otherwise specified, all compared schemes use the same settings.

The target-aware blockage prediction network is trained in a supervised manner using the generated link-state labels. Each training instance contains a short multi-view image sequence and the corresponding target vehicle template. The main training parameters are summarized in Table 2.

To evaluate the computational feasibility of the proposed predictor, the model is implemented using Python 3.10.20, PyTorch 2.3.1, and CUDA 12.1, and its inference latency is measured on an NVIDIA GeForce RTX 4090 GPU (NVIDIA Corporation, Santa Clara, CA, USA) with a batch size of one. The average inference latency is 5.52 ms per sample with a standard deviation of 1.13 ms, and the 95th-percentile latency is 7.03 ms. These values are substantially shorter than the nominal 0.5-s look-ahead interval corresponding to H=1, indicating that the prediction can be completed within the considered slot-level operation time.

### 5.3. Baseline Schemes and Evaluation Metrics

To evaluate the proposed framework, we compare both vision-based blockage prediction and communication-level relay triggering performance with representative baselines.

For the main communication-level evaluation, the UAV position is fixed at $q_{U}={\left(-1,\;4.5,\;60\right)}^{T}$ m. Its horizontal projection corresponds to the point on the road centerline closest to the RSU, which is selected as a representative geometric reference for relaying between the stationary RSU and the moving vehicle. The same position is used for all compared relay-triggering schemes.

For vision-based blockage prediction, representative baselines covering different visual-input, target-association, multimodal-fusion, and temporal-modeling strategies are considered. The single-camera baseline uses only one camera view while keeping the same temporal prediction setting. The GPS-guided association baseline uses external position information to associate the target vehicle with visual observations. In addition, a GPS–image fusion baseline combines visual features with target-position information for future link-state prediction, while the Transformer-based baseline retains the same target-aware multi-view visual front end as the proposed method but replaces the LSTM temporal module with a lightweight Transformer encoder. These baselines provide comparisons across visual-input, target-association, multimodal-fusion, and temporal-modeling strategies under the same future link-state prediction task.

For the vision-prediction results reported in Table 3 and Table 4, each model configuration is independently trained n=3 times under the same fixed scene-sequence-level train–validation split, and split-level variability is not evaluated.

In addition, component-level ablation experiments are conducted under the nominal prediction horizon H=1 to examine the roles of the target template, template–scene interaction, dual-view observations, CBAM, and temporal modeling. In each ablation variant, only the component under investigation is removed or replaced, while the remaining architecture and training settings are kept unchanged. For the “without template” variant, the template feature is replaced by an all-zero tensor, thereby removing target-specific template information. For the “without correlation” variant, both the projected scene and template features are retained, but their element-wise multiplicative interaction is removed. Specifically, instead of concatenating the projected scene feature with the template-conditioned feature obtained through element-wise multiplication, the broadcast template feature is directly concatenated with the projected scene feature. Therefore, the input dimensionality of the subsequent fusion layer and all downstream modules remains unchanged. The two single-view variants use Camera 1 and Camera 2 individually, respectively, while the “without LSTM” variant replaces the LSTM-based temporal module with direct feature concatenation followed by a fully connected classifier.

For communication-level evaluation, four relay triggering schemes are compared. The RSU-only scheme uses only the direct RSU–vehicle link without UAV relay assistance. The reactive UAV relay scheme activates the UAV only after an NLoS blockage has been detected. A nominal response delay of 0.5 s is introduced between blockage detection and relay activation to represent detection, signaling, and activation latency, while its sensitivity is further evaluated over 0–2 s. The proposed predictive UAV relay scheme triggers the UAV according to the predicted NLoS probability and the selected operating threshold. The oracle UAV relay scheme uses the ground-truth link state at each slot for relay triggering and serves as a performance reference.

Vision prediction performance is evaluated by prediction accuracy, and the training loss is reported to show convergence behavior. For communication-level performance, Absent samples are excluded from evaluation, and the outage probability and UAV activation ratio follow the definitions in Section 3. In addition, two reliability-oriented rate metrics are adopted to evaluate service quality under blockage-prone conditions.

The 5th-percentile rate is used to characterize lower-tail service quality:

(44) $$R_{5\%}={\mathrm{Percentile}}_{5}\left(\left\{R\left(t\right)|t\in T_{\mathrm{eff}}\right\}\right).$$

A higher $R_{5\%}$ indicates better rate robustness under unfavorable channel conditions.

To characterize blockage mitigation capability, the NLoS average rate is calculated over the slots where the direct RSU–vehicle link is in the NLoS state:

(45) $${\bar{R}}_{\mathrm{NLoS}}=\frac{1}{|T_{\mathrm{NLoS}}|}\sum_{t\in T_{\mathrm{NLoS}}}R\left(t\right),$$

where $T_{\mathrm{NLoS}}=\left\{t\in T_{\mathrm{eff}}|s_{t}=1\right\}$. This metric reflects the effectiveness of UAV relay triggering during blockage-prone periods.

Together, these metrics evaluate prediction accuracy, lower-tail service quality, blockage-period rate performance, outage reduction, and UAV relay usage.

## 6. Simulation Results

### 6.1. Vision-Based Blockage Prediction Performance

This subsection evaluates the visual blockage prediction performance of the proposed target-aware multi-camera method. The evaluation includes comparisons with representative visual-association and temporal-modeling baselines, together with prediction accuracy, NLoS-oriented classification metrics, convergence behavior, GPS-localization sensitivity, prediction-horizon sensitivity, and auxiliary mutual-information analysis.

**Figure 5:**
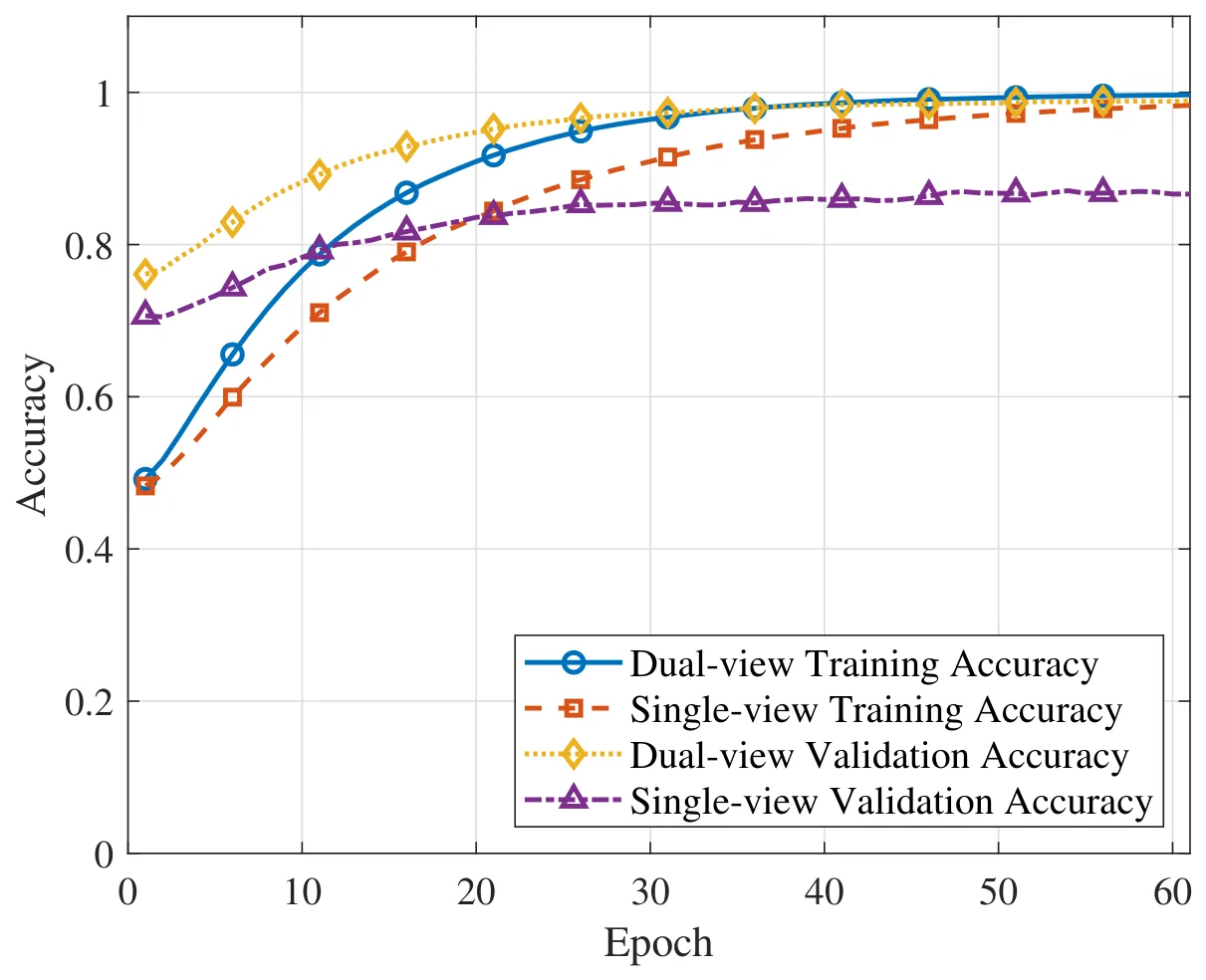
Prediction accuracy comparison between single-view and dual-view visual prediction.

Figure 5 compares the training and validation accuracy of the single-view and dual-view prediction models. The dual-view model achieves about 99% validation accuracy after convergence, whereas the single-view model converges to approximately 87%, yielding an improvement of about 12 percentage points. This result indicates that the two camera views provide complementary visual cues for identifying the target vehicle and potential blockers, thereby reducing view-dependent uncertainty in future blockage prediction.

**Figure 6:**
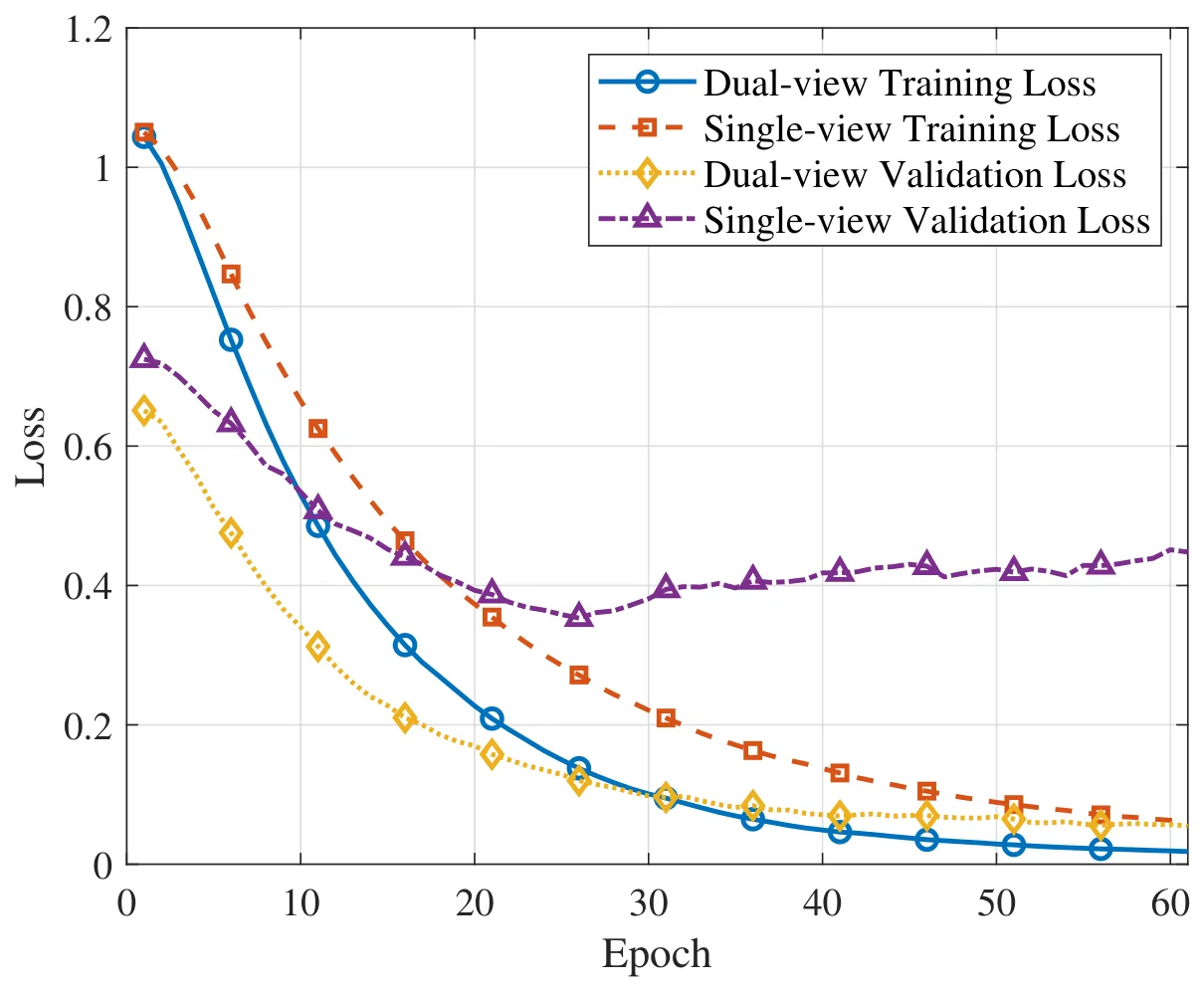
Loss comparison between single-view and dual-view visual prediction.

Figure 6 shows the loss curves of the two prediction models. Although both models converge during training, the dual-view model achieves a much lower validation loss. Specifically, the validation loss of the dual-view model decreases to about 0.06, whereas that of the single-view model remains around 0.45. This observation is consistent with the accuracy comparison in Figure 5, confirming that multi-view observations provide more discriminative information for learning future blockage states.

**Figure 7:**
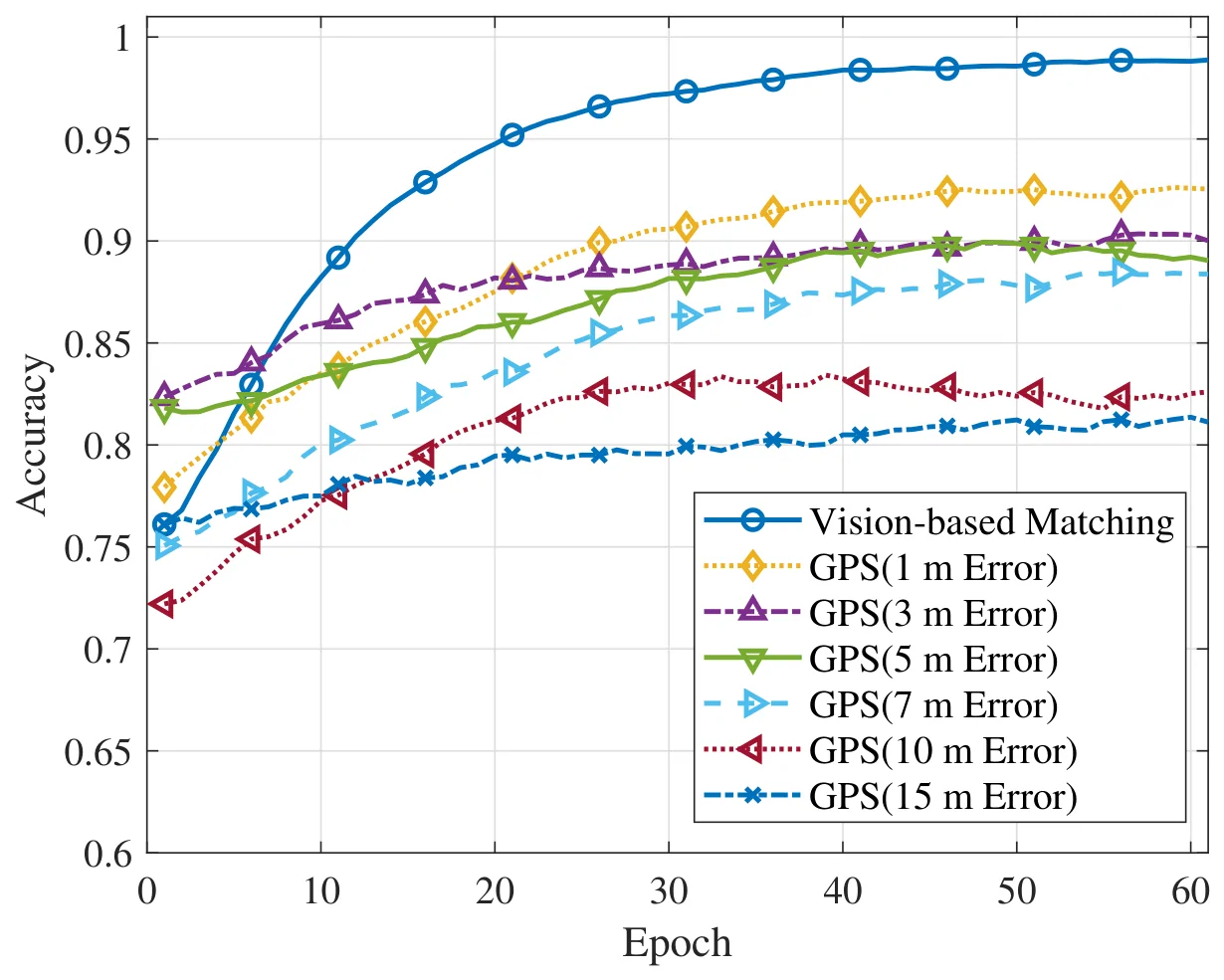
Prediction accuracy comparison between GPS-guided association and image-matching-based target association.

Figure 7 compares the proposed image-matching-based association with GPS-guided association under different localization errors. The proposed method maintains about 99% prediction accuracy. In contrast, the GPS-guided baseline achieves about 92–93% accuracy when the localization error is small, but drops to approximately 81–83% when the error increases to 10–15 m. In this high-error region, the proposed method outperforms the GPS-guided baseline by about 16–18 percentage points. The performance degradation of the GPS-guided baseline is mainly caused by coordinate-to-image mapping errors, which may associate the target vehicle with an incorrect visual region in dense traffic scenes. Since the proposed method performs target association directly in the visual feature space, it avoids such cross-modal mapping errors and achieves more robust target-aware blockage prediction. Beyond overall prediction accuracy, proactive relay triggering depends particularly on identifying future NLoS events. We therefore next examine the precision–recall characteristics of the NLoS prediction probability used for relay activation.

Table 3 compares the proposed method with a Transformer-based temporal predictor and a GPS–image fusion baseline. For the nominal horizon H=1, all three methods achieve similar overall performance, while the proposed method obtains the highest average accuracy and Macro-F1 and maintains a competitive NLoS F1 score. At the longer horizon H=8, prediction becomes more challenging for all methods. The GPS–image fusion baseline achieves the highest average performance on several metrics, while the proposed method remains competitive and provides higher accuracy, Macro-F1, NLoS recall, and NLoS F1 than the Transformer-based baseline. These results indicate that the proposed target-aware multi-view architecture provides competitive blockage-prediction performance across both short and relatively long prediction horizons. Having established the competitiveness of the proposed predictor against stronger temporal-modeling and multimodal baselines, we next examine the robustness of its target-association mechanism to localization uncertainty.

**Figure 8:**
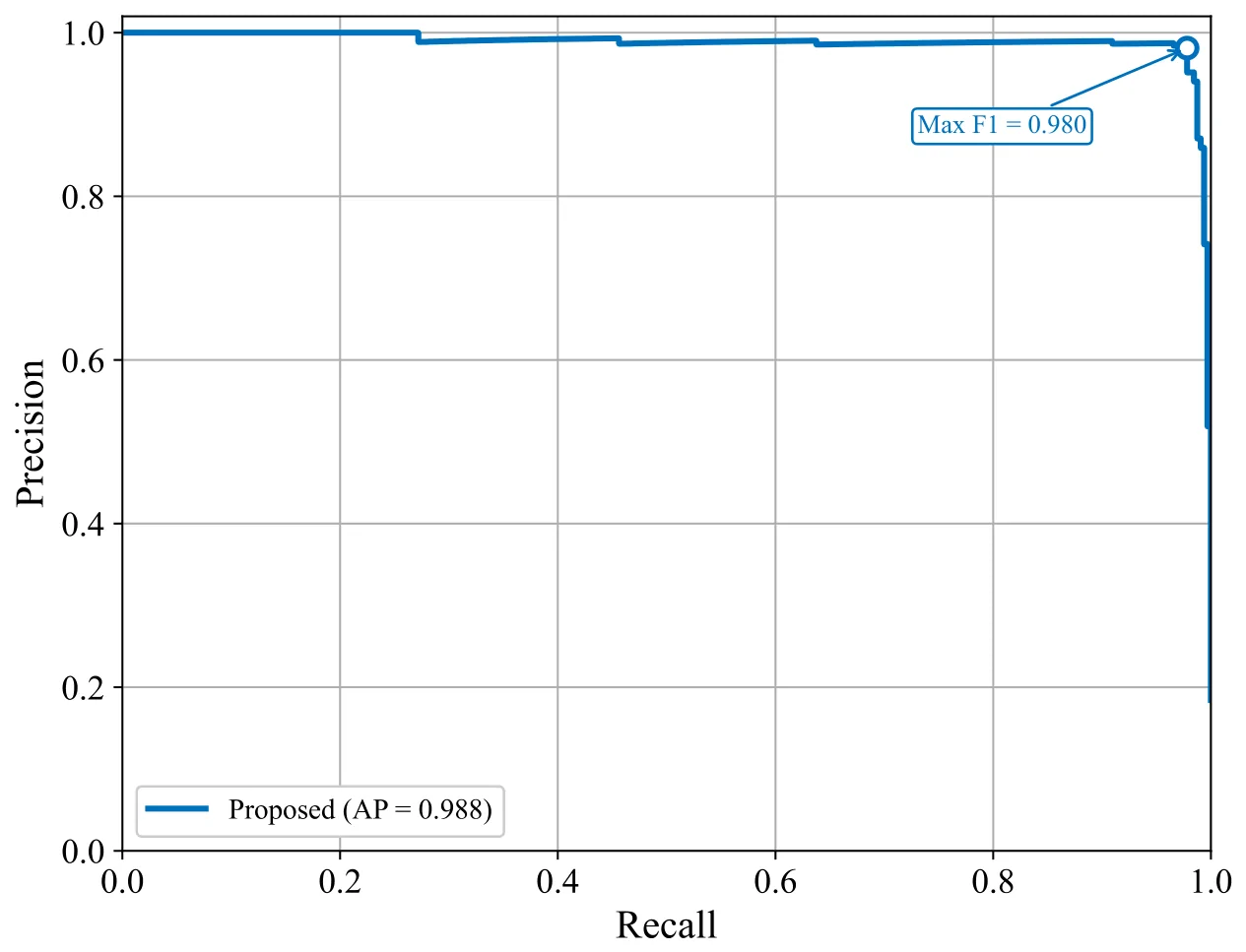
Precision–recall curve for NLoS prediction on the validation set. The marked point indicates the operating threshold selected by maximizing the NLoS F1 score.

Figure 8 shows the precision–recall curve of the proposed predictor for the NLoS class. The curve is obtained by varying the NLoS-probability threshold on the validation set. A lower threshold generally increases recall at the expense of precision, whereas a higher threshold produces the opposite tradeoff. The operating threshold is selected at the point that maximizes the NLoS F1 score and is then fixed for the subsequent evaluations.

After characterizing the operating behavior of the trained predictor, we further isolate the contributions of its major components through a component-level ablation study under the nominal horizon H=1. The corresponding results are summarized in Table 4. The same dataset split, prediction horizon, and training settings are used across the ablation variants, with only the component under investigation removed or replaced.

The ablation results first demonstrate the importance of target-specific template information and its interaction with the scene features. Removing the informative target template causes the largest performance degradation, reducing the accuracy from 98.23% to 59.33% and the NLoS F1 from 95.90% to 39.37%. When both the template and scene features are retained but their element-wise multiplicative interaction is removed, the accuracy and NLoS F1 decrease to 90.84% and 85.23%, respectively. Importantly, this variant preserves both types of input features as well as the input dimensionality of the subsequent fusion module. Therefore, the observed degradation specifically demonstrates the benefit of the template-conditioned multiplicative interaction rather than simply reflecting the removal of template information or a reduction in the downstream model size.

The single-view variants also exhibit substantial degradation. Using Camera 1 alone yields an NLoS F1 of 59.53%, while using Camera 2 alone yields 78.37%, compared with 95.90% for the full dual-view model. The different degradation patterns of the two single-view variants further indicate that the two roadside views provide complementary target-related information, supporting the use of multi-view feature fusion.

**Figure 9:**
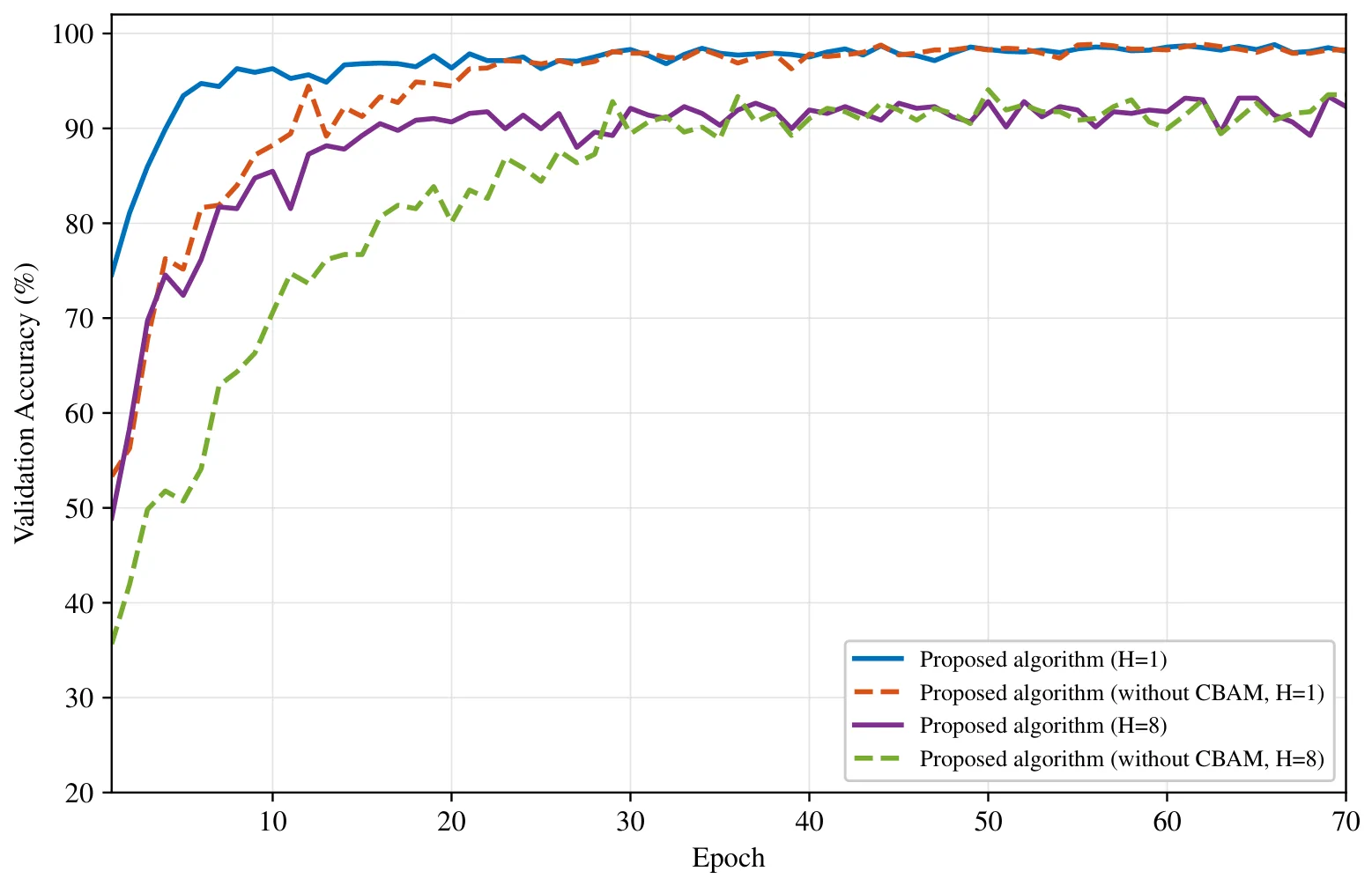
Validation-accuracy convergence of the proposed predictor with and without CBAM under H=1 and H=8.

As shown in Figure 9, removing CBAM has little effect on the saturated validation accuracy, which is consistent with the final metrics in Table 4. However, the model without CBAM exhibits slower convergence during the early training stage under both prediction horizons. This result suggests that, in the considered setting, CBAM mainly facilitates feature refinement and training optimization rather than providing a substantial improvement in the final saturated classification accuracy.

The contribution of temporal modeling depends more strongly on the prediction horizon. Under the nominal H=1 setting, replacing the LSTM with direct feature concatenation followed by a fully connected classifier produces final metrics close to those of the full model, indicating that the current visual observations already provide strong cues for one-slot-ahead prediction. At the longer horizon H=8, however, the full model achieves an accuracy of 92.32%, a Macro-F1 of 89.93%, and an NLoS F1 of 78.99%, whereas the corresponding values without the LSTM decrease to 90.44%, 87.78%, and 76.02%, respectively. These results suggest that explicit temporal modeling becomes more useful as the prediction interval increases and future blockage states depend more strongly on the temporal evolution of the observed traffic scene.

Overall, the ablation results distinguish the roles of the main architectural components: target–template interaction and dual-view observations primarily affect target-specific prediction performance, whereas CBAM and temporal modeling mainly influence training behavior and longer-horizon prediction, respectively. Having examined the component-level contributions, we next evaluate how the complete predictor behaves as the prediction horizon itself is varied.

**Figure 10:**
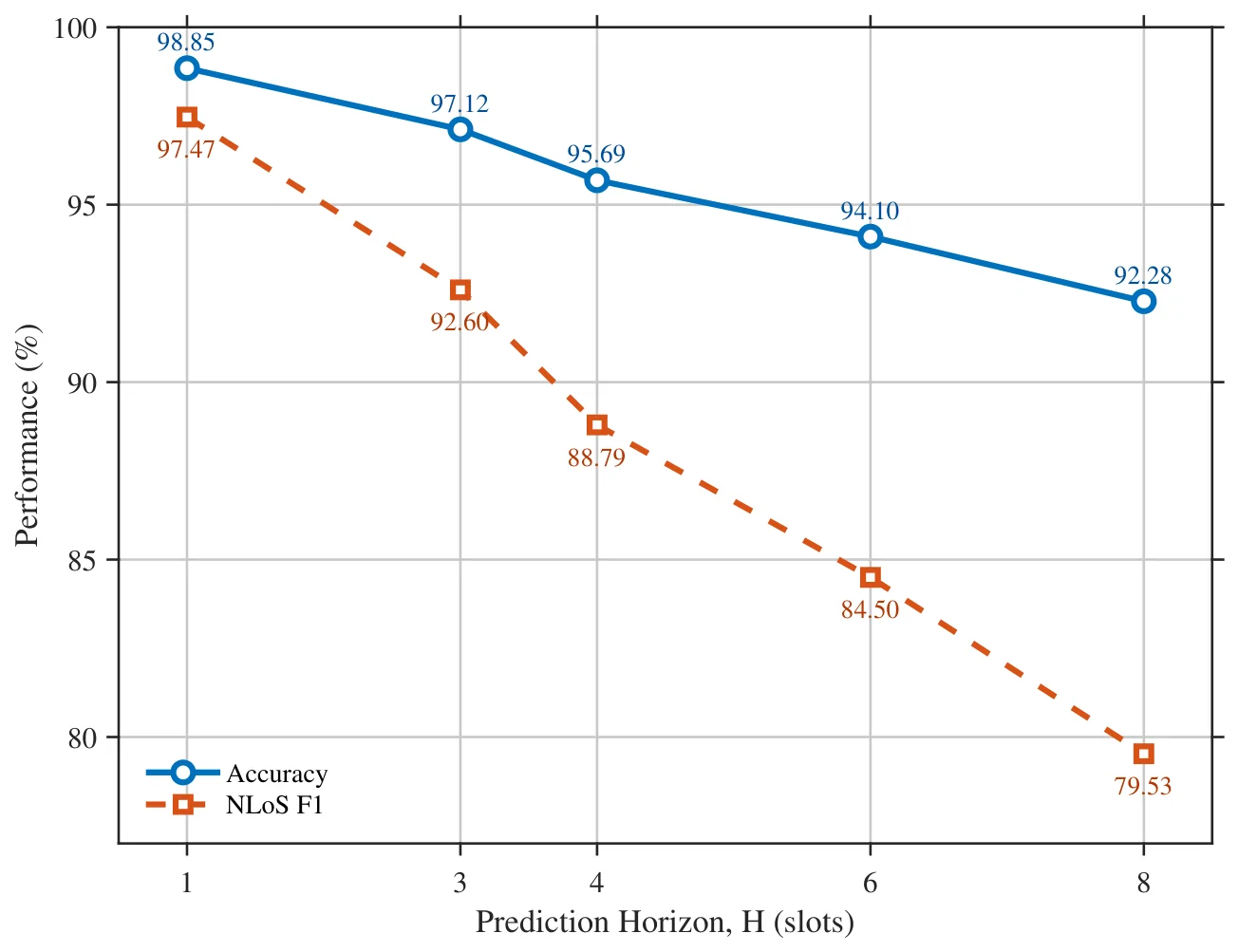
Sensitivity of blockage prediction performance to the prediction horizon *H*.

Figure 10 evaluates the prediction performance under representative horizons H∈{1,3,4,6,8}, corresponding to look-ahead intervals from 0.5 s to 4 s. For each horizon, the model is independently retrained using the same architecture and training settings. Both the accuracy and NLoS F1 decrease as the prediction horizon increases, with a more pronounced degradation in NLoS F1, indicating the higher difficulty of anticipating future blockage over longer intervals. These results reveal a tradeoff between prediction performance and proactive response time; therefore, H=1 is adopted as the nominal setting in the main experiments. Finally, since the preceding ablation results show a clear benefit from dual-view input, we use mutual-information analysis as an auxiliary tool to interpret how the information contributions of the two camera views vary across different link states.

Table 5 reports the mutual-information values of the two camera views under different target-link states. Although the absolute differences between the two views are relatively small, a consistent state-dependent pattern can be observed across the total, gray-level, and color information measures. Camera 1/RSU provides slightly higher mutual information under LoS conditions, whereas Camera 2 provides higher mutual information under NLoS conditions; for the Absent state, the two views exhibit similar values. This behavior is consistent with the different performance degradation observed for the two single-view variants in Table 4. Therefore, the mutual-information analysis is used as auxiliary evidence that the two roadside viewpoints contain complementary state-dependent visual cues, rather than as evidence of a large absolute information difference between the cameras.

### 6.2. UAV Relay Triggering Performance

**Figure 11:**
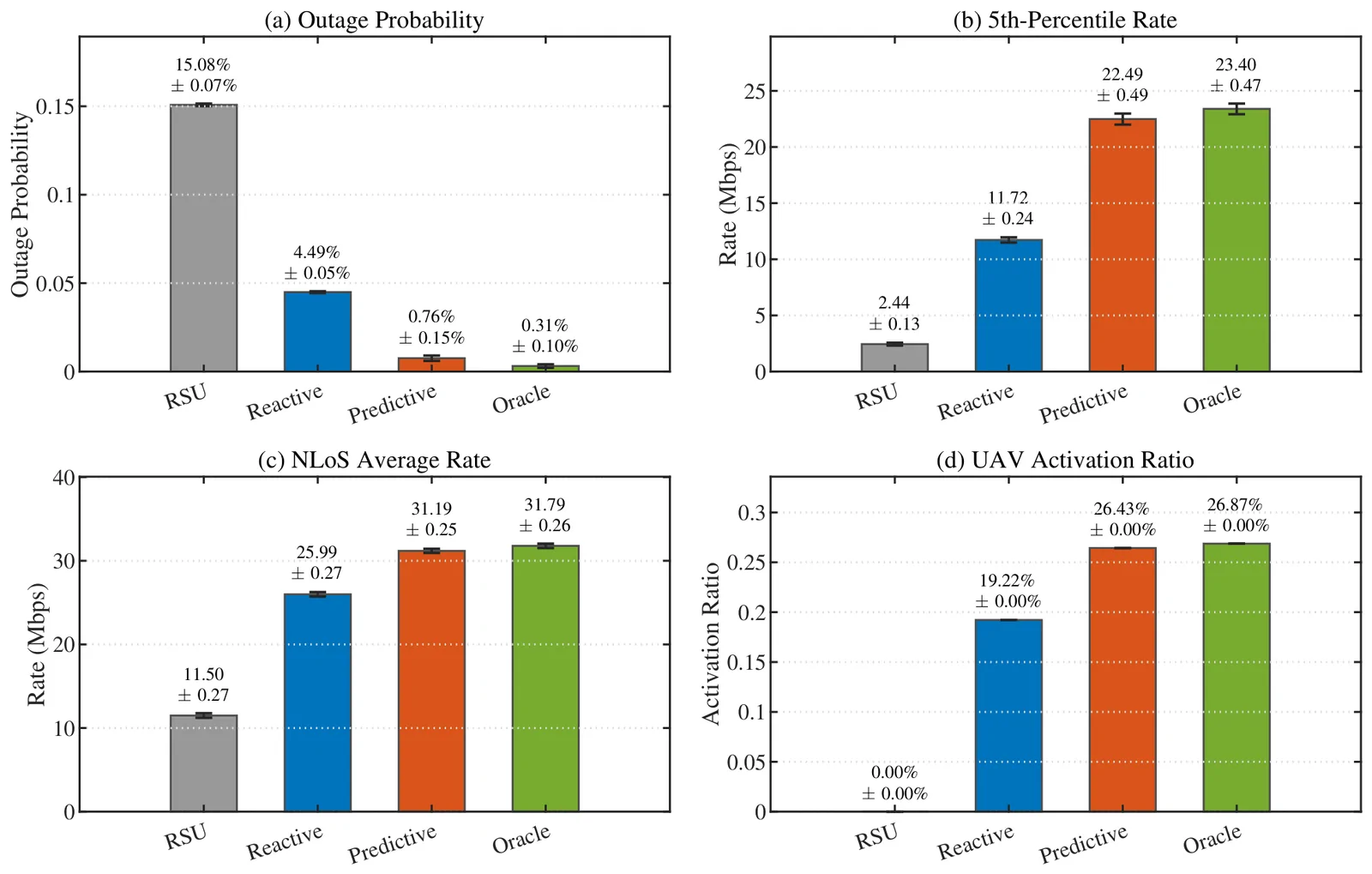
Communication performance comparison of different relay triggering schemes.

This subsection evaluates the communication-level performance of the proposed prediction-assisted UAV relay triggering scheme. The RSU-only, reactive UAV relay, proposed predictive UAV relay, and oracle UAV relay schemes are compared in terms of outage probability, 5th-percentile rate, NLoS average rate, and UAV activation ratio. The main performance comparison is shown in Figure 11. Since the overall average rate can be dominated by LoS samples and may not fully reflect blockage-period performance, the following analysis focuses on outage probability, 5th-percentile rate, and NLoS average rate.

As shown in Figure 11, the RSU-only scheme has the highest outage probability of 15.08%, because it cannot provide an alternative transmission path when the direct link becomes unreliable. The reactive UAV scheme reduces the outage probability to 4.49% by activating the UAV after NLoS blockage is detected, but its delayed response still causes service degradation during fast blockage transitions. In contrast, the proposed predictive scheme further reduces the outage probability to 0.76%. Relative to the oracle mean outage probability of 0.31%, the proposed scheme shows a mean difference of 0.45 percentage points.

The rate-related metrics show a similar trend. Compared with the reactive scheme, the proposed scheme increases the 5th-percentile rate from 11.72 Mbps to 22.49 Mbps. Compared with the oracle mean value of 23.40 Mbps, the corresponding mean difference is 0.91 Mbps, or approximately 3.9%. During ground-truth NLoS slots, the proposed scheme achieves an average rate of 31.19 Mbps, compared with 31.79 Mbps for the oracle scheme, corresponding to a mean difference of 0.60 Mbps, or approximately 1.9%. These differences are modest and should be interpreted in the context of the reported variability.

The above results establish the nominal communication-level benefit of predictive relay triggering. The following analyses further examine whether this conclusion is sensitive to relay-link propagation, triggering operating point, prediction quality, reactive response latency, and UAV placement. To evaluate the robustness to non-ideal A2G propagation, we extend the nominal LoS-dominant relay-link model by allowing the RSU–UAV and UAV–vehicle hops to independently experience LoS or NLoS conditions. Each hop is LoS with probability $P_{\mathrm{LoS}}$, which is varied over {0.7,0.8,0.9,1.0}. When an A2G hop is NLoS, an additional excess attenuation is applied to its large-scale path loss, while the remaining communication parameters are kept unchanged.

**Figure 12:**
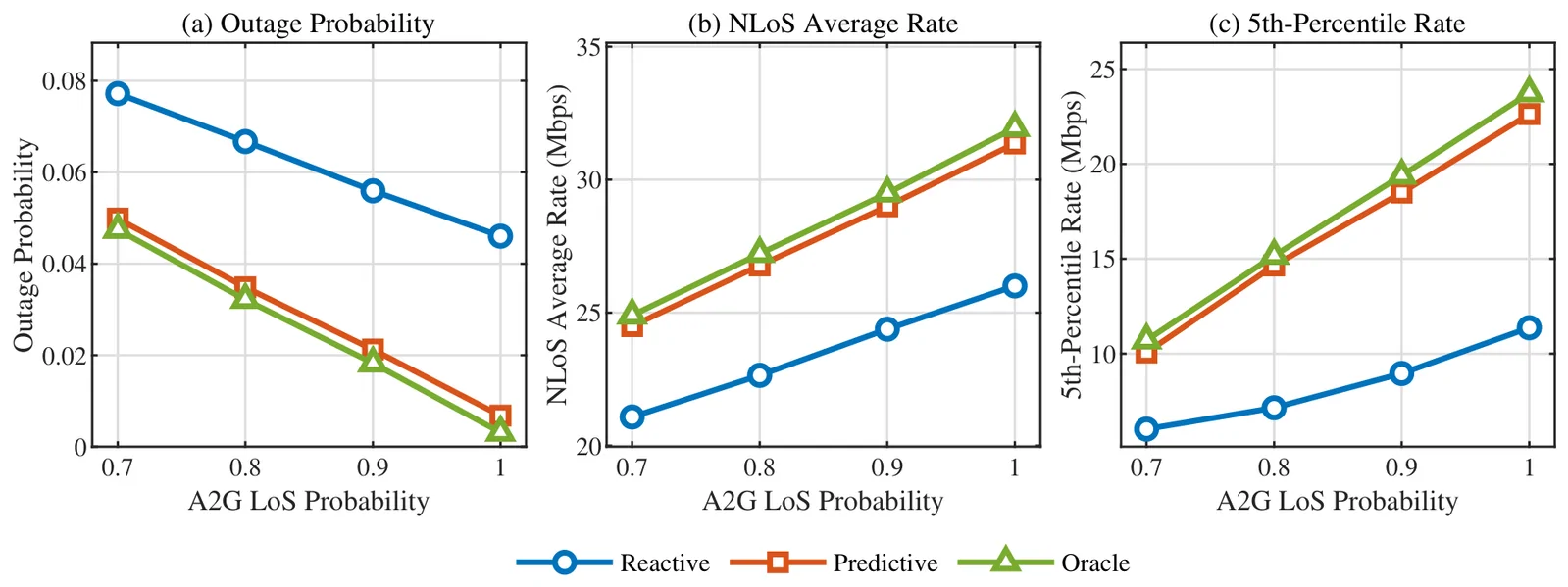
Communication-performance sensitivity to the A2G LoS probability under probabilistic LoS/NLoS relay-link conditions.

As shown in Figure 12, decreasing $P_{\mathrm{LoS}}$ increases the outage probability and reduces both the NLoS average rate and the 5th-percentile rate for all UAV-assisted schemes. Nevertheless, the predictive scheme consistently outperforms the reactive scheme over the considered range, indicating that its performance advantage is preserved when the relay links are no longer assumed to remain continuously LoS.

**Figure 13:**
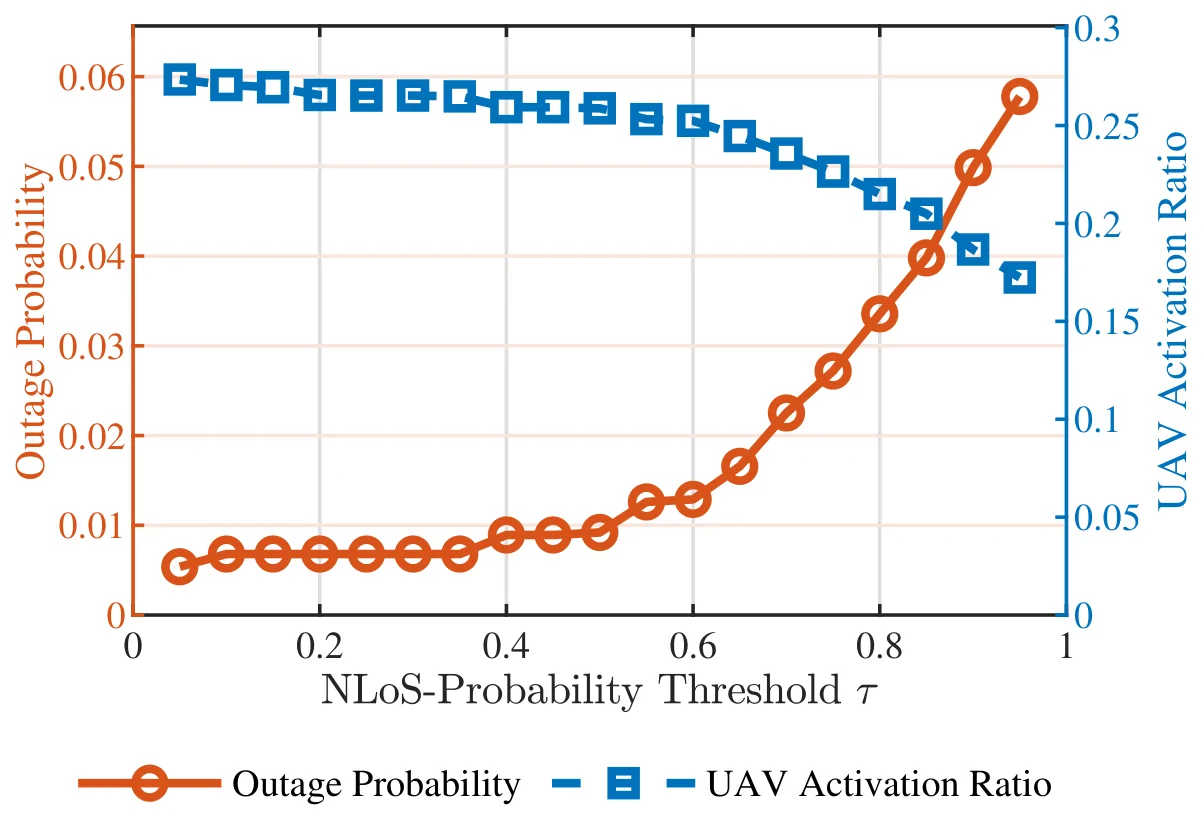
Communication-level tradeoff between outage probability and UAV activation ratio under different NLoS-probability thresholds.

Figure 13 evaluates the communication-level impact of the NLoS-probability threshold. At relatively low thresholds, the UAV is activated more aggressively, and the outage probability remains low. As τ increases, the UAV activation ratio gradually decreases, whereas the outage probability increases, particularly at higher thresholds. This behavior reveals a clear reliability–relay-usage tradeoff and demonstrates that the probability-based triggering policy provides an adjustable operating point instead of relying on a fixed hard-class decision.

**Figure 14:**
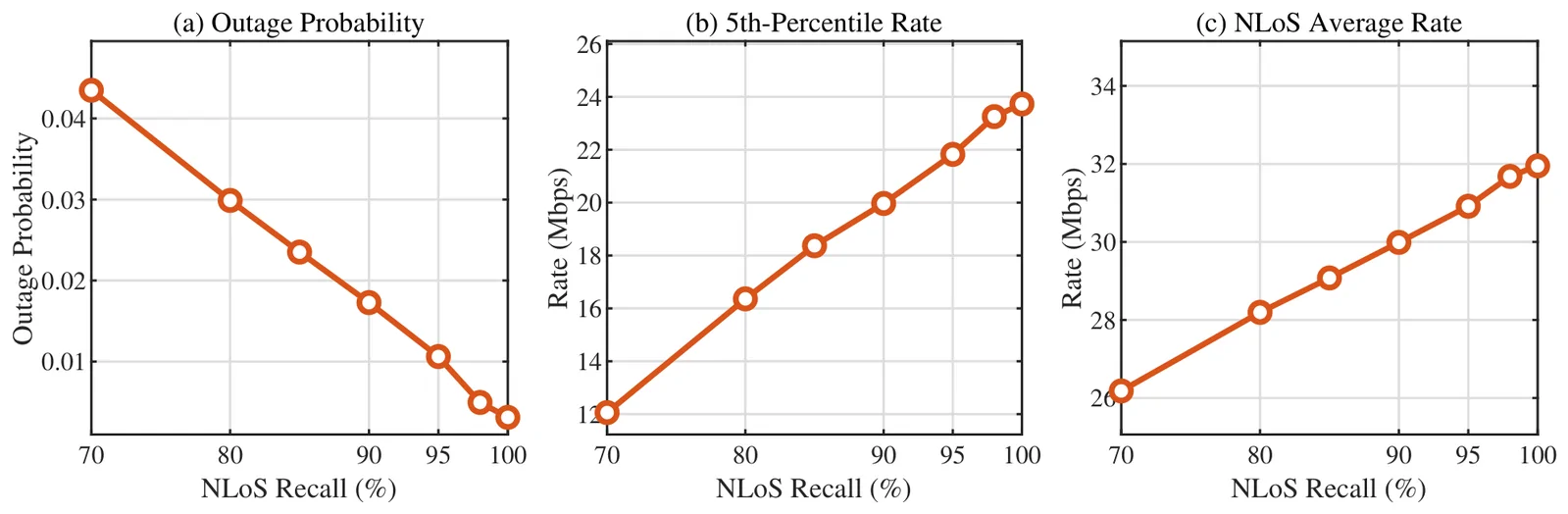
Sensitivity of communication performance to NLoS recall.

Complementary to the actual precision–recall characteristics in Figure 8, Figure 14 provides a controlled system-level sensitivity analysis of how different NLoS recall levels affect communication performance. As the NLoS recall increases, the outage probability decreases substantially, while both the 5th-percentile rate and the NLoS average rate increase. These results confirm that detecting future NLoS events is critical for proactive relay triggering, because missed NLoS predictions may leave the UAV inactive during blockage-prone periods. Therefore, Figure 8 characterizes the precision–recall operating points of the trained predictor, whereas Figure 14 isolates the communication-level sensitivity to missed NLoS events.

When the NLoS recall approaches 100%, the additional performance gain becomes relatively small. This indicates that the framework is more sensitive to missed NLoS events in the lower-recall region. Once most future NLoS events can be detected in advance, the remaining prediction errors have a relatively limited impact on communication performance.

**Figure 15:**
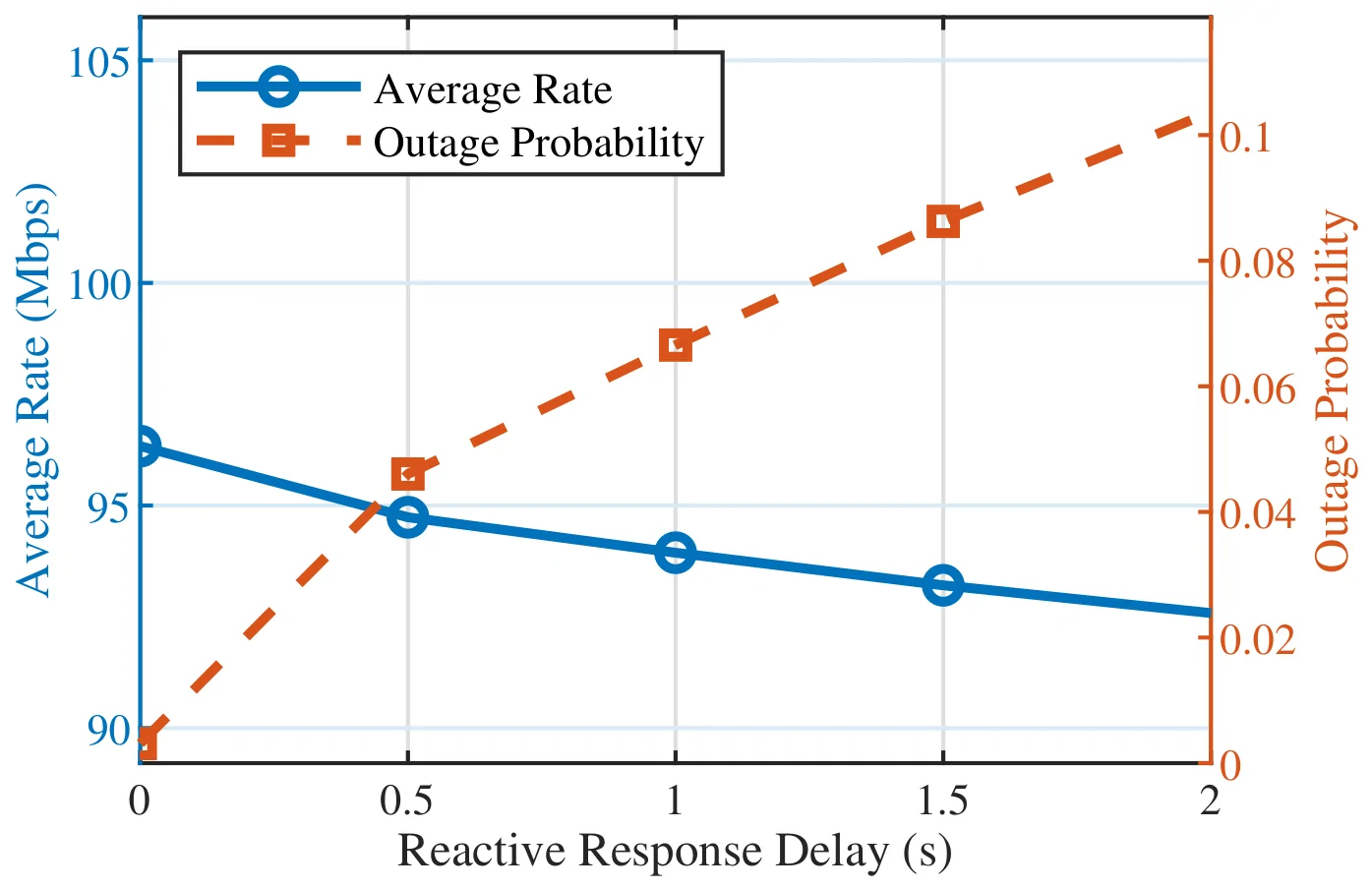
Sensitivity of the reactive UAV relay scheme to response delay.

To examine whether the comparison depends on the assumed reactive response latency, Figure 15 evaluates the Reactive scheme over delays from 0 to 2 s. The zero-delay case represents immediate activation after blockage detection, whereas larger delays account for additional detection, signaling, and activation latency. The results confirm that reactive performance is sensitive to this latency, while the full sweep avoids relying on the nominal 0.5 s setting alone. In addition to timing assumptions, the quality of the relay path also depends on the UAV geometry. We therefore conclude the sensitivity analysis by examining the effect of UAV horizontal placement.

**Figure 16:**
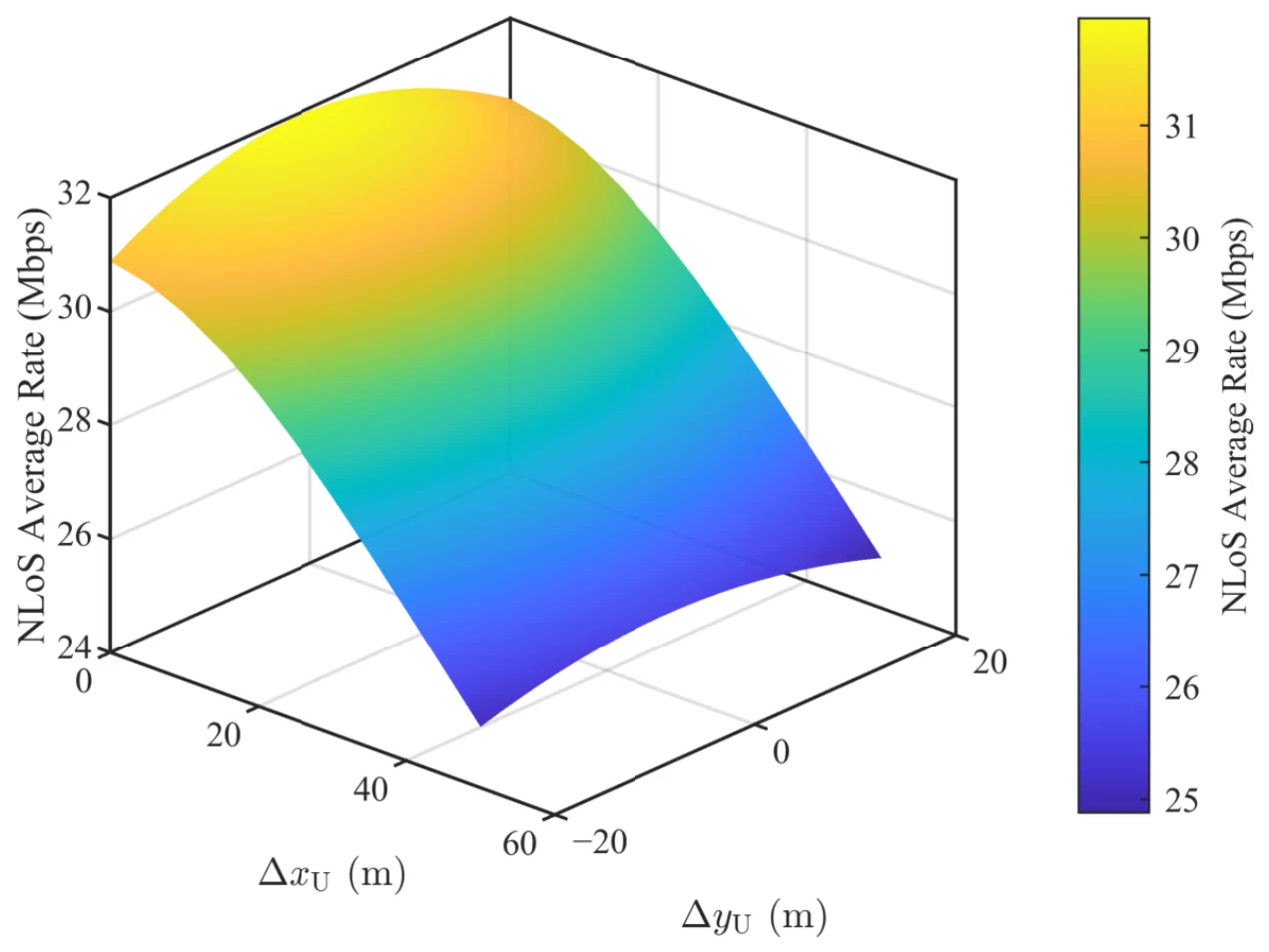
Sensitivity of NLoS average rate to UAV horizontal placement.

Figure 16 shows the sensitivity of the NLoS average rate to UAV horizontal placement. Since the performance variation along the road direction is nearly symmetric, only the nonnegative longitudinal displacement $|\Delta x_{U}|$ is shown. The NLoS average rate ranges from about 25 Mbps to over 31 Mbps, indicating a clear dependence on UAV placement. A favorable position improves the two-hop air–ground link budget and yields a higher blockage-period rate, whereas an unfavorable position reduces the relay gain. Notably, all considered UAV placements provide substantially higher NLoS average rates than the RSU-only benchmark shown in Figure 11. The nominal UAV position used in the main comparison is selected as a representative geometric reference, with its horizontal projection located at the point on the road centerline closest to the RSU, rather than through UAV placement optimization.

Taken together, these sensitivity analyses provide a broader assessment of the communication-level robustness of the proposed framework under variations in A2G propagation, triggering operation, prediction quality, reactive response latency, and UAV placement.

## 7. Conclusions

This paper proposed a vision-assisted UAV relay triggering framework for proactive blockage mitigation in air–ground integrated mmWave V2X networks. The framework leverages roadside multi-camera perception to predict the future LoS, NLoS, and Absent states of the target RSU–vehicle link and uses the predicted NLoS probability to determine the UAV DF relay triggering decision through a probability-based policy. This enables relay assistance before severe direct-link degradation occurs.

Simulation results show that the proposed dual-view predictor achieves about 99% validation accuracy, outperforming the single-view baseline with about 87% accuracy. At the communication level, the proposed predictive relay scheme reduces the outage probability from 15.08% for RSU-only transmission and 4.49% for reactive relaying to 0.76%. It also improves the 5th-percentile rate from 11.72 Mbps to 22.49 Mbps over reactive relaying. Compared with the oracle reference, the corresponding mean differences are 0.45 percentage points in outage probability, 0.91 Mbps in the 5th-percentile rate, and 0.60 Mbps in the NLoS average rate. These differences are modest and should be interpreted in the context of the reported variability. These results demonstrate that target-aware visual prediction provides useful prior information for proactive UAV relay triggering and enhances the reliability of blockage-prone mmWave V2X links.

The current evaluation is conducted within a fixed road geometry and camera deployment and therefore reflects within-scene rather than cross-scene generalization; evaluating cross-scene generalization under different road layouts and sensing configurations remains an important direction for future work. A future direction is to extend the proposed perception–prediction–relay framework from the considered RSU–vehicle link to low-altitude urban air–ground networks. In such scenarios, blockage prediction should cover not only terrestrial RSU–vehicle links but also air–ground and air-to-air links involving UAV nodes. Since urban buildings, roadside objects, and dynamic obstacles may degrade the links of both ground vehicles and low-altitude aerial nodes, a unified visual blockage prediction mechanism can be developed to identify potential LoS interruptions across ground and aerial links. Based on such prediction, UAV relay assistance can be extended from supporting blocked ground vehicles to serving aerial nodes with degraded links, thereby improving the robustness of low-altitude air–ground communications in obstacle-rich urban environments.

## Figures and Tables

**Table 1:** Main communication simulation parameters.

Parameter	Value
Carrier frequency $f_{c}$	30 GHz
System bandwidth *B*	20 MHz
RSU transmit power $P_{R}$	23 dBm
UAV transmit power $P_{U}$	20 dBm
Number of RSU antennas $N_{R}$	16
UAV altitude	60 m
Slot duration Δt	0.5 s
Prediction horizon *H*	1 slot (0.5 s)
Reactive response delay	0.5 s
Noise power density	−174 dBm/Hz
Noise figure $F_{N}$	7 dB
Implementation loss $L_{\mathrm{imp}}$	5 dB
Maximum spectral efficiency $\eta_{\max}$	6 bps/Hz
LoS/NLoS path-loss exponents $\alpha_{L}/\alpha_{N}$	2.1/3.5
Additional NLoS loss $L_{N}$	20 dB
Nakagami-*m* for LoS/NLoS/A2G links	3/1/2
Outage threshold $R_{\mathrm{th}}$	10 Mbps
A2G LoS probability $P_{\mathrm{LoS}}$	0.7–1.0 (robustness only)
A2G NLoS excess attenuation	15 dB (robustness only)

**Table 2:** Network training parameters.

Parameter	Value
Input frame number *r*	3
Scene image size	480×270×3
Target template size	68×34×3
Batch size	32
Training epochs	60
Optimizer	Adam
Initial learning rate	$1\times 10^{-3}$
Learning-rate decay factor	0.95
Weight decay	$1\times 10^{-4}$

**Table 3:** Comparison with representative alternative blockage prediction methods under different prediction horizons. Results are reported as mean ± standard deviation over three independent runs.

Method	*H*	Accuracy (%)	Macro-F1 (%)	NLoS Precision (%)	NLoS Recall (%)	NLoS F1 (%)
Proposed	1	98.23±0.53	97.69±0.86	97.68±1.43	94.22±2.53	95.90±1.20
Transformer-based	1	98.00±0.50	97.58±0.66	98.15±0.61	93.23±2.66	95.61±1.13
GPS–image fusion	1	98.04±0.89	97.51±1.40	96.60±1.95	95.42±5.56	95.92±2.32
Proposed	8	92.32±0.70	89.93±0.75	74.96±3.31	83.78±5.03	78.99±0.52
Transformer-based	8	89.18±2.98	85.44±3.35	75.69±1.67	70.80±5.36	73.07±2.97
GPS–image fusion	8	93.54±0.76	91.37±0.49	74.09±5.61	86.73±7.02	79.59±0.66

**Table 4:** Component-level ablation results under the nominal prediction horizon H=1. Results are reported as mean ± standard deviation across repeated training runs.

Configuration	Accuracy (%)	Macro-F1 (%)	NLoS F1 (%)
Without template	59.33±0.00	37.73±0.14	39.37±0.45
Without correlation	90.84±2.61	88.07±3.24	85.23±3.71
Camera 1 only	88.13±1.14	79.30±2.66	59.53±6.45
Camera 2 only	82.63±0.45	81.74±0.31	78.37±0.04
Without CBAM	98.21±0.57	97.74±0.98	96.43±1.99
Without LSTM	98.04±0.24	97.71±0.29	96.56±0.66
Proposed	98.23±0.53	97.69±0.86	95.90±1.20

**Table 5:** Mutual-information values of the two camera views under different target-link states.

Metric	State	Camera 1/RSU	Camera 2
Total	LoS	2.102	2.047
	NLoS	2.029	2.142
	Absent	2.016	2.013
Gray	LoS	2.096	2.053
	NLoS	2.033	2.149
	Absent	2.018	2.022
Color	LoS	2.106	2.039
	NLoS	2.024	2.132
	Absent	2.011	2.001

## Data Availability

The data and implementation code used in this study are not publicly available due to confidentiality and data-management restrictions associated with an industry-collaborative research project.
